# Lipoprotein Receptor LRP1 Regulates Leptin Signaling and Energy Homeostasis in the Adult Central Nervous System

**DOI:** 10.1371/journal.pbio.1000575

**Published:** 2011-01-11

**Authors:** Qiang Liu, Juan Zhang, Celina Zerbinatti, Yan Zhan, Benedict J. Kolber, Joachim Herz, Louis J. Muglia, Guojun Bu

**Affiliations:** 1 Department of Pediatrics, Washington University School of Medicine, St. Louis, Missouri, United States of America; 2 Department of Molecular Genetics, University of Texas Southwestern Medical Center, Dallas, Texas, United States of America; 3 Department of Cell Biology and Physiology, Washington University School of Medicine, St. Louis, Missouri, United States of America; 4 Department of Neuroscience, Mayo Clinic, Jacksonville, Florida, United States of America; 5 Institute for Biomedical Research, Xiamen University, Xiamen, China; University of Cambridge, United Kingdom

## Abstract

Obesity is a growing epidemic characterized by excess fat storage in adipocytes. Although lipoprotein receptors play important roles in lipid uptake, their role in controlling food intake and obesity is not known. Here we show that the lipoprotein receptor LRP1 regulates leptin signaling and energy homeostasis. Conditional deletion of the *Lrp1* gene in the brain resulted in an obese phenotype characterized by increased food intake, decreased energy consumption, and decreased leptin signaling. LRP1 directly binds to leptin and the leptin receptor complex and is required for leptin receptor phosphorylation and Stat3 activation. We further showed that deletion of the *Lrp1* gene specifically in the hypothalamus by Cre lentivirus injection is sufficient to trigger accelerated weight gain. Together, our results demonstrate that the lipoprotein receptor LRP1, which is critical in lipid metabolism, also regulates food intake and energy homeostasis in the adult central nervous system.

## Abstract 2

Lipoprotein receptor LRP1 play critical roles in lipid metabolism, and this study reveals a novel role for LRP1 in controlling food intake and obesity in the central nervous system of the adult mouse.

## Author Summary

The World Health Organization estimates that at least 1 in 10 adults worldwide are obese, and in some western countries, a far greater percentage (25% or more) is affected. Obesity is a serious concern because it increases the risk of cardiovascular disease, type 2 diabetes, and some cancers, among other health problems. Despite recent advances in understanding the disease mechanism, effective treatments are still lacking. Lipoprotein receptors play critical roles in lipid metabolism, but their potential roles in controlling food intake and obesity in the central nervous system have not been examined. Here we show that deletion of LRP1, a member of the LDL (low density lipoprotein) receptor family, in the adult mouse brain results in obese phenotype characterized by increased food intake, decreased energy consumption and decreased leptin signaling. We further show that deletion of the *Lrp1* gene specifically in the hypothalamus (a region of the brain) by using Cre lentivirus injection is sufficient to trigger accelerated weight gain. Together, our results present a novel function of LRP1: the direct regulation of leptin signaling and energy balance in the adult central nervous system. Hence, LRP1 represents a very promising new therapeutic target for the design of innovative and more effective therapies for obesity.

## Introduction

The low-density lipoprotein receptor-related protein 1 (LRP1) is a large cell surface receptor ubiquitously expressed in a variety of organs including adipose tissue, liver, and brain [1]. Previous tissue-specific knockout studies showed that hepatic LRP1 mediates the metabolism of apolipoprotein E (apoE)-rich chylomicron remnants [2] and that adipocyte LRP1 modulates postprandial lipid transport and glucose homeostasis [3]. Furthermore, LRP1 has a pivotal role in preventing atherosclerosis by restricting smooth muscle cell proliferation and protecting vascular wall integrity [4]. In the central nervous system (CNS), LRP1 is highly expressed in neurons and plays critical roles in lipoprotein metabolism, neurotransmission, synaptic plasticity, cell survival, and clearance of the amyloid-β (Aβ) peptide, critical in the pathogenesis of Alzheimer's disease (AD) [5]–[7].

## Results and Discussion

### Neuronal Deletion of *Lrp1* in the Adult Mouse Brain Leads to Obesity

To investigate the roles of LRP1 in the adult CNS, we generated conditional *Lrp1* forebrain knockout mice (LRP1-KO) by crossing *Lrp1* floxP mice [2] with αCamKII-Cre mice [8]. Because αCamKII-Cre is only expressed in neurons of the adult brain [8], the essential function of LRP1 during embryonic development [9] is preserved. *Lrp1* deletion in different brain regions was assessed by comparing LRP1 protein expression levels between LRP1-KO (*Lrp1^flox+/+^/Cre^+/^* ^−^, LRP1 knockout) and WT (*Lrp1^flox+/+^/Cre* ^−*/*−^, *Lrp1* floxP littermate control) mice at 3, 6, 9, and 12 mo of age. LRP1 expression was not significantly decreased in the LRP1-KO mice at 3 mo of age. However, from 6 to 12 mo of age, LRP1 expression was decreased by ∼75% in the cortex, hypothalamus, and hippocampus of LRP1-KO mice (Figure S1A–S1C). The residual LRP1 observed in these regions likely represents LRP1 expressed in glial cells [10]. Inactivation of the *Lrp1* gene was specific to the forebrain, as no changes in LRP1 expression were detected in the cerebellum or in peripheral tissues (Figure S1D and S1E).

**Figure 1:**
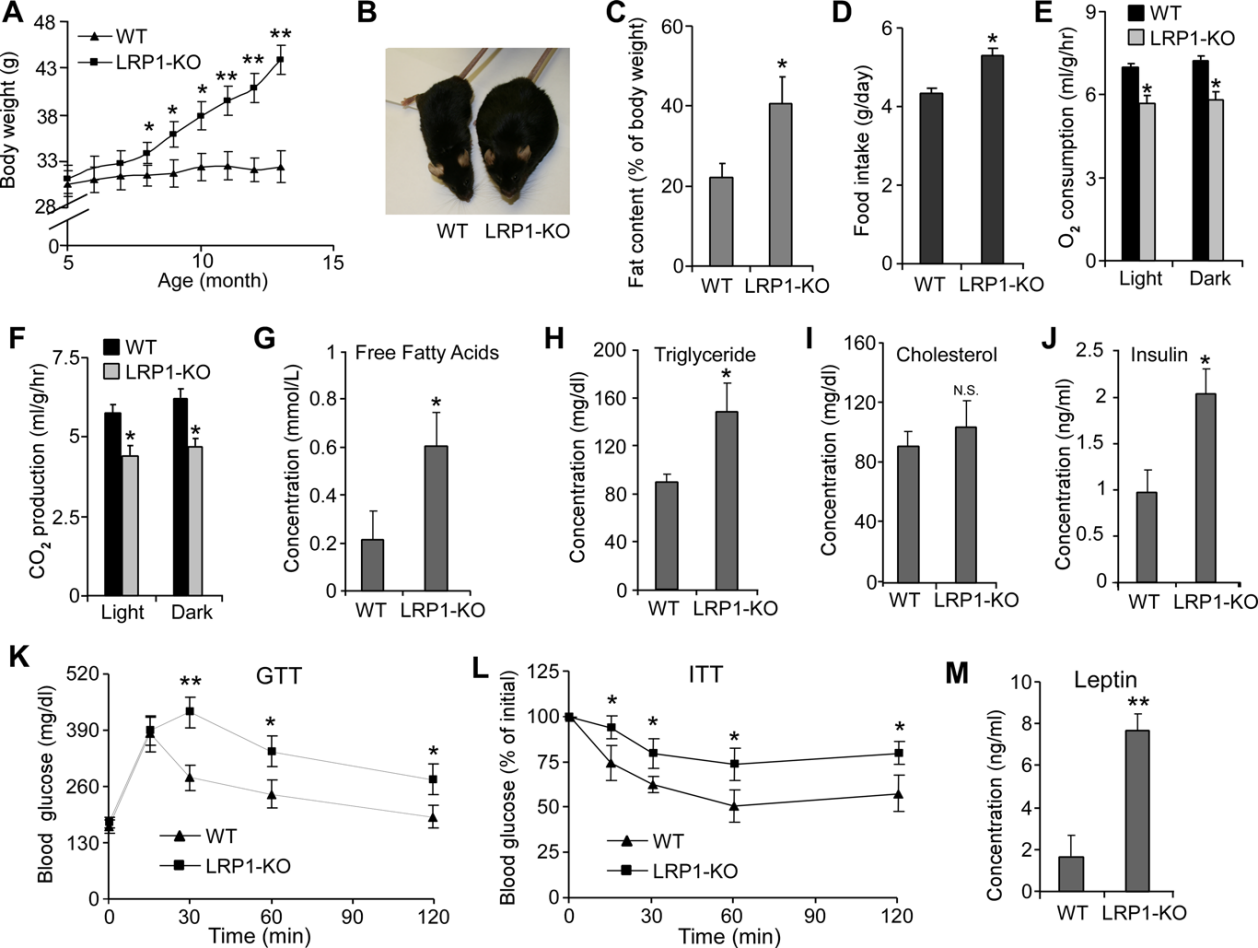
Neuronal deletion of *Lrp1* in the adult mouse brain leads to obesity associated with hyperlipidemia, glucose intolerance, and insulin resistance. (A) Body weights of male LRP1-KO (*Lrp1^flox+/+^/Cre^+/^* ^−^, LRP1 knockout) and WT (*Lrp1^flox+/+^/Cre* ^−*/*−^, *Lrp1* floxp littermate control) mice were determined at the indicated ages (*n* = 10, *p<0.05; **p<0.01). (B) A representative LRP1-KO mouse and a WT mouse at 12 mo of age showing the obese phenotype associated with LRP1-KO. (C) Fat content in whole body assessed by magnetic resonance imaging (MRI) in LRP1-KO and WT mice at 12 mo of age (*n* = 8, *p<0.05). (D) Food intake of LRP1-KO and WT mice at 9 mo of age was determined daily over a 1-wk period (*n* = 7, *p<0.05). (E, F) Energy expenditure of LRP1-KO and WT mice at 13 mo of age was measured by O_2_ consumption (E) and CO_2_ production (F) during both light cycle and dark cycle (*n* = 6, *p<0.05). (G–J) Blood samples were obtained from overnight-fasted LRP1-KO and WT mice at 12 mo of age and levels of free fatty acids (G), triglyceride (H), cholesterol (I), and insulin (J) were determined. For (G–J), *n* = 9, *p<0.05; N.S., not significant. (K) Glucose tolerance tests were performed on LRP1-KO and WT mice at 12 mo of age following 16 h of fasting (*n* = 6, *p<0.05). (L) Insulin tolerance tests were performed on LRP1-KO and WT mice at 12 mo of age following 6 h of fasting (*n* = 7, *p<0.05). (M) Plasma leptin levels were determined by ELISA on blood samples obtained from LRP1-KO and WT mice at 12 mo of age (*n* = 10, *p<0.05). Error bars are mean ± s.e.m.

LRP1-KO mice were apparently indistinguishable from control littermates during the first 6 mo of life but showed significantly accelerated body weight gain starting at 7 mo of age (Figure 1A and S2A). This increased weight gain in LRP1-KO mice correlated closely with the observed decrease in LRP1 expression in the CNS. On a normal chow diet, LRP1-KO mice became obese at about 12 mo of age (Figure 1B) and had approximately 2-fold increased body fat content compared to their littermate controls (Figure 1C). No changes in snout-anus length between LRP1-KO mice and WT mice were observed (Figure S2B). LRP1-KO mice ate significantly more than controls (Figure 1D) and had significantly decreased energy expenditure, as revealed by decreased O_2_ consumption and CO_2_ production (Figure 1E and 1F). Together, these results indicated that LRP1 expression in the brain controls body weight and adiposity by regulating food intake and energy expenditure.

Obesity in LRP1-KO mice was associated with hyperlipidemia and insulin resistance. LRP1-KO mice showed a 3-fold increase in circulating free fatty acids (FFA) and ∼50% increase in circulating triglycerides (TG) (Figure 1G and 1H). Plasma cholesterol levels were not significantly altered in LRP1-KO mice (Figure 1I). At about 12 mo of age, LRP1-KO mice showed approximately 2-fold increase in plasma insulin levels (Figure 1J). While fasting blood glucose levels appeared to be normal (unpublished data), LRP1-KO mice had significantly decreased tolerance to exogenous glucose when assessed by intraperitoneal glucose tolerance test (GTT). A marked increase in both magnitude and duration of blood glucose in response to glucose injection was observed in LRP1-KO mice (Figure 1K). LRP1-KO mice also failed to reduce blood glucose levels after insulin injection (insulin tolerance test, ITT) (Figure 1L). Thus, the obese phenotype in LRP1-KO mice was characterized by hyperlipidemia and insulin resistance, resembling the metabolic syndrome pathology in humans.

### LRP1 Regulates Leptin Signaling

**Figure 2:**
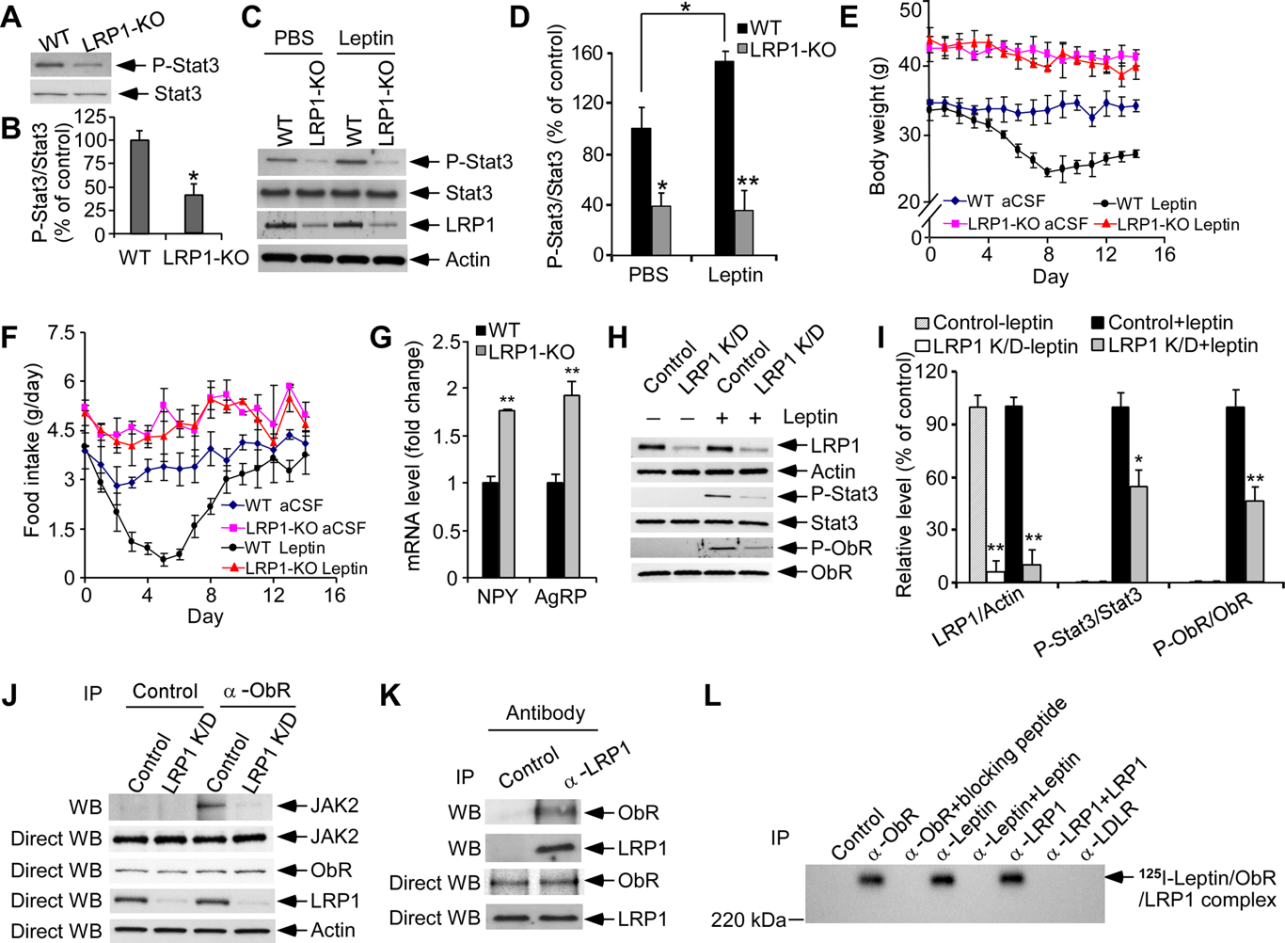
LRP1 regulates leptin signaling. (A) Hypothalamic phosphorylated Stat3 (P-Stat3) was decreased in neuronal LRP1-KO mice. Levels of P-Stat3 and total Stat3 in the hypothalamus of LRP1-KO and WT mice at 13 mo of age were evaluated by Western blotting. An equal amount of sample protein was loaded in each lane in this and subsequent figures. (B) Densitometric quantification of P-Stat3 and total Stat3 levels was performed as described in Materials and Methods (*n* = 4, *p<0.05). (C) Deletion of LRP1 in adult brain led to decreased leptin sensitivity. LRP1-KO and WT mice at 12 mo of age were injected intraperitoneally with leptin (1 mg/kg body weight) or PBS as control. Hypothalamic extracts were prepared 45 min after injection. Levels of LRP1, P-Stat3, and total Stat3 were analyzed by Western blotting. (D) Densitometric quantification of P-Stat3 and total Stat3 levels was performed as described in Materials and Methods (*n* = 4, *p<0.05; **p<0.01). (E, F) Effect of ICV leptin or artificial cerebrospinal fluid (aCSF) infusion on body weight (E) and food intake (F) in LRP1-KO and WT mice at 12 mo of age (*n* = 7). (G) Hypothalamic expression of genes encoding neuropeptides was compared between LRP1-KO and WT mice at 13 mo of age (*n* = 4, *p<0.05; **p<0.01; N.S., not significant) by quantitative real-time RT-PCR. (H) GT1-7 cells were transiently transfected with control siRNA or LRP1-specific siRNA for 48 h, serum-starved overnight, and then treated with 50 nM leptin or control for 30 min. Levels of LRP1, P-Stat3, total Stat3, and ObR were analyzed by Western blotting. For P-ObR, extracts from GT1-7 cells were first immunoprecipitated with an anti-phosphotyrosine antibody and then immunoblotted with an anti-ObR antibody. (I) Densitometric analysis of Western blot samples (*n* = 4, *p<0.05; **p<0.01) indicates that knockdown of LRP1 greatly decreased the ratio of P-Stat3/Stat3 and the ratio of P-ObR/ObR. (J) GT1-7 cells were transiently transfected with control siRNA or LRP1-specific siRNA for 48 h. Extracts were prepared from GT1-7 cells and immunoprecipitated with either a control antibody or an anti-ObR antibody followed by immunoblotting with an anti-JAK2 antibody. Extracts were also directly immunoblotted with the ObR antibody, JAK2 antibody, and LRP1 antibody. (K) Extracts were prepared from GT1-7 cells and immunoprecipitated with either a control antibody or an anti-LRP1 antibody followed by immunoblotting with an anti-ObR antibody. Extracts were also directly immunoblotted with the anti-ObR antibody and anti-LRP1 antibody. (L) Ligand binding was performed by incubating GT1-7 cells with ^125^I-leptin for 1 h at 4°C. Chemical crosslinking was then carried out, followed by immunoprecipitation with control antibody, anti-LRP1 antibody, anti-LRP1 antibody + full-length LRP1 protein, anti-leptin antibody, anti-leptin antibody + leptin protein, anti-ObR antibody, anti-ObR antibody + specific blocking peptide, or anti-LDLR antibody, and immunoprecipitates were analyzed on SDS-PAGE. Error bars are mean ± s.e.m.

Increase in fat storage observed in LRP1-KO mice was accompanied by approximately 5-fold increase in plasma leptin concentrations (Figure 1M). Because leptin secreted by adipose tissue plays a major role in body weight regulation [11]–[13], we hypothesized an existing leptin-resistant condition in LRP1-KO mice. To directly assess a role for LRP1 in leptin signaling we measured the levels of phospho-Stat3 (P-Stat3) [14] in the hypothalamus of LRP1-KO and WT mice. A significant decrease in the levels of P-Stat3 was observed in LRP1-KO mice (Figure 2A, 2B and S2C, S2D). In addition, deletion of LRP1 in the adult brain led to leptin insensitivity as shown by impaired leptin-stimulated phosphorylation of hypothalamic Stat3 (Figure 2C and 2D). Central leptin sensitivity was also evaluated by introcerebroventricular (ICV) leptin administration. Chronic ICV infusion of leptin caused a rapid reduction in body weight (Figure 2E) and food intake (Figure 2F) in WT control mice but had minimal effects on LRP1-KO mice (Figure 2E and 2F), suggesting that LRP1 deletion led to leptin resistance.

In the arcuate nucleus of the hypothalamus, leptin regulates energy imbalance by inhibiting expression of orexigenic neuropeptide Y (NPY) and agouti-related protein (AgRP) [13]–[17]. We found that deletion of LRP1 markedly increased NPY and AgRP mRNA levels in the hypothalamus (Figure 2G). Further, using immunofluorescence staining, we demonstrated that LRP1 colocalized with AgRP but not with POMC (Figure S3), suggesting that LRP1 may specifically regulate leptin signaling in AgRP neurons. These results are consistent with compromised leptin signaling in the hypothalamus of LRP1-KO mice and further support leptin signaling as the potential mechanism by which LRP1 regulates food intake and energy expenditure in the adult mice.

To further elucidate the molecular and cellular mechanisms underlying LRP1 regulation of leptin signaling we used GT1-7 cells, a neuronal cell line derived from the mouse hypothalamus [18]. LRP1 knockdown in GT1-7 cells by LRP1-specific siRNA significantly decreased the phosphorylation of both Stat3 and leptin receptor ObR (Figure 2H, 2I and S4A). While treatment of control GT1-7 cells with leptin dramatically increased the phosphorylation of both Stat3 and ObR, it only had lesser effects on LRP1 knockdown cells (Figure 2H, 2I and S5). In addition, we found that LRP1 knockdown significantly reduced the phosphorylation of the extracellular signal-regulated kinase (ERK), which is also downstream to the leptin receptor signaling (Figure S4B and S4C) [19]. Previous studies have shown that the leptin receptor mediates Stat3 phosphorylation via janus kinase 2 (JAK2) activation [13]–[15]. Using co-immunoprecipitation, we confirmed that LRP1 knockdown in GT1-7 cells decreased the interaction between ObR and JAK2 (Figure 2J, S6A, S6B, S6D, and S7A). We also found that LRP1 knockdown decreased leptin-mediated phosphorylation of JAK2 (Figure S7B and S7C).

Because LRP1 had been previously shown to serve as co-receptor to PDGF signaling via the PDGF receptor [4], we next examined the potential for a direct association between LRP1 and the leptin receptor. Using GT1-7 cellular extracts, we found that the LRP1 antibody, but not a control antibody, co-immunoprecipitated the leptin receptor ObR in the presence of leptin but not in the absence of leptin (Figure 2K, S6C, and S6E). To further confirm direct binding of LRP1 to the leptin/leptin receptor complex, we followed binding of ^125^I-leptin to GT1-7 cells with chemical cross-linking [20]. Under these conditions, radiolabeled leptin migrated as a high molecular weight complex on SDS-PAGE that was immunoreactive with leptin, leptin receptor, and LRP1 antibodies, but not with a control antibody or an antibody to the LDL receptor (LDLR) (Figure 2L, S6F, and S8). The specificity of each antibody in these co-immunoprecipitation experiments was confirmed by antigen blocking. Together, these results suggest that LRP1 regulates leptin signaling by forming a complex with leptin and the leptin receptor.

### Hypothalamic LRP1 Regulates Food Intake and Energy Homeostasis

**Figure 3:**
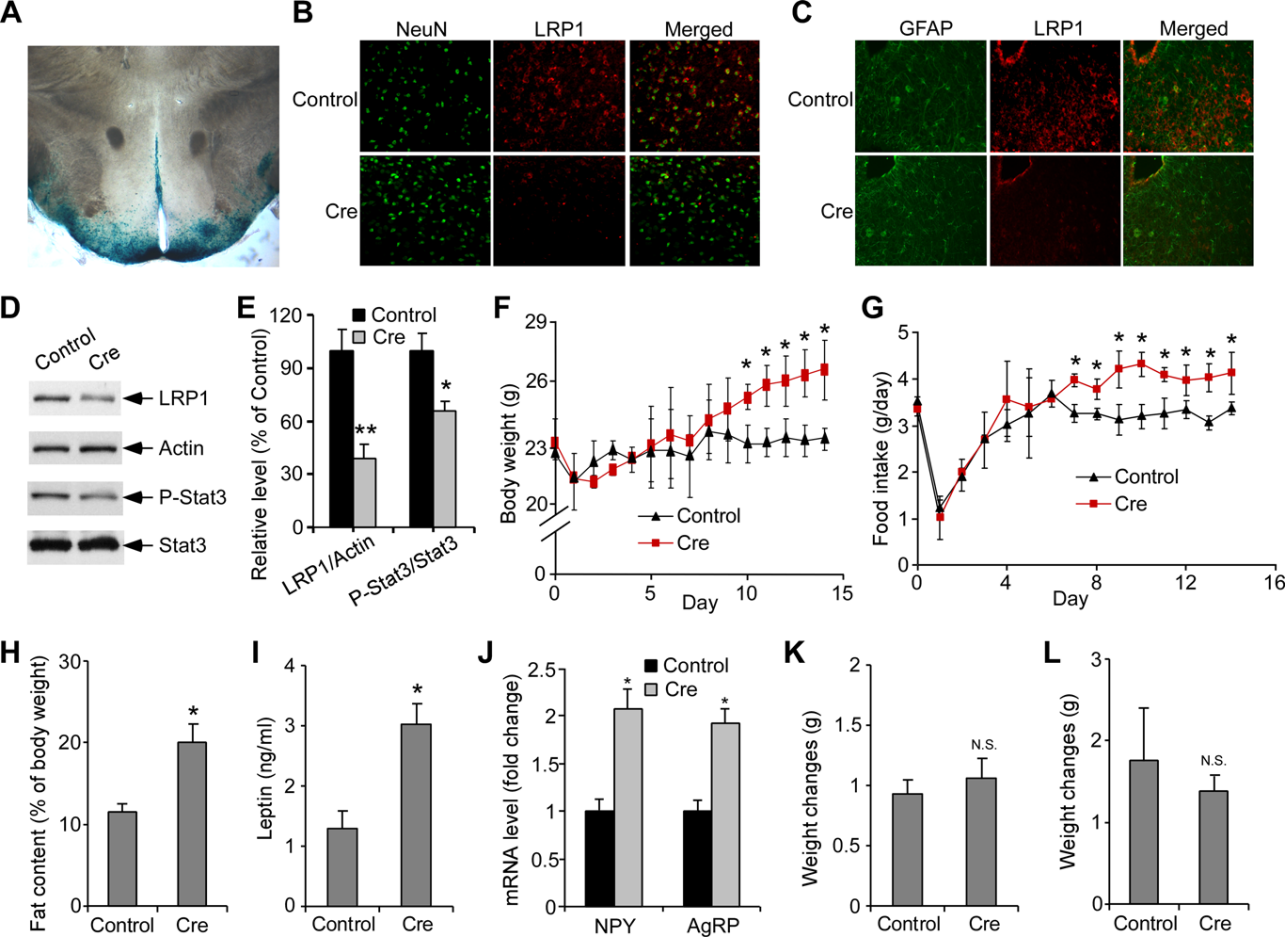
Hypothalamic LRP1 regulates leptin signaling and body energy homeostasis. (A) Representative image demonstrating the hypothalamus lentivirus injection technique. Arcuate nucleus of the hypothalamus of Rosa-26 reporter mice was bilaterally injected with Cre lentivirus and then stained for LacZ. (B–J) *Lrp1* floxp mice (*Lrp1^flox+/+^/Cre* ^−*/*−^) at 8–10 wk of age were bilaterally injected with either Cre lentivirus or control GFP lentivirus. Fourteen days after lentivirus injection, (B,C) double immunofluorescence staining was performed using either an anti-LRP1 antibody (detected with Alexa 568, red) and anti-NeuN antibody (detected with Alexa 488, green) (B) or an anti-LRP1 antibody (detected with Alexa 568, red) and anti-GFAP antibody (detected with Alexa 488, green) (C). A representative staining in ARC of hypothalamus is shown. (D) Levels of LRP1, P-Stat3, total Stat3, and actin in the hypothalamus were analyzed by Western blotting. (E) Densitometric analyses of Western blot samples (*n =* 4, *p < 0.05; **p < 0.01) indicate that LRP1 deletion in the hypothalamus significantly decreased the ratio of P-Stat3/Stat3. (F, G) Body weight (F) and food intake (G) were measured after lentivirus injection (*n* = 5, *p < 0.05). (H) Fat content in whole body was compared between GFP control and Cre lentivirus injected mice (*n  = * 4, *p < 0.05) by MRI. (I, J) Fourteen days after lentivirus injection, (I) plasma leptin levels were determined by ELISA on blood samples (*n* = 6, *p<0.05). (J) Hypothalamic expression of genes encoding neuropeptides was compared between Control and Cre lentivirus injection mice (*n* = 6, *p<0.05; N.S., not significant) by quantitative real-time PCR. (K) *Lrp1* floxp mice were injected bilaterally into the cortex with either Cre lentivirus or control GFP lentivirus (*n* = 5, N.S., not significant). Body weight changes were measured 14 d after the lentivirus injection. (L) Wild-type mice (C57BL/6) were injected bilaterally into the arcuate nucleus of hypothalamus with either Cre lentivirus or control GFP lentivirus (*n* = 5, N.S., not significant). Body weight changes were measured 14 d after the lentivirus injection. Error bars are mean ± s.e.m.

The hypothalamus is central to the control of food intake and energy expenditure; therefore LRP1 deletion in the hypothalamus of LRP1-KO mice is likely the major contributor to the obese phenotype. To further test this hypothesis, we deleted the *Lrp1* gene specifically in the hypothalamus using lentivirus delivery technology (Figure 3A). Direct injection of Cre lentivirus into the arcuate nucleus of the hypothalamus of *Lrp1* floxp mice resulted in significant neuronal LRP1 deletion, as measured by immunofluorescence and Western blotting (Figure 3B–3E and S9) and reduction of leptin signaling, as measured by reduced levels of P-Stat3 (Figure 3D and 3E). Strikingly, Cre lentivirus-injected *Lrp1* floxp mice also showed significantly greater body weight gain, increased food intake, and higher fat content compared to control GFP lentivirus-injected mice (Figure 3F–3H). Plasma levels of leptin (Figure 3I) were significantly increased in Cre lentivirus-injected mice. In addition, Cre lentivirus-injected mice had markedly increased expression of NPY and AgRP (Figure 3J). Cre lentivirus-injected mice also showed leptin insensitivity as detected by impaired leptin-stimulated phosphorylation of hypothalamic Stat3 (Figure S10A and S10B). As controls, Cre lentivirus injection into either the cortical region of the *Lrp1* floxp mice or the arcuate nucleus of the hypothalamus in wild-type mice (C57BL/6) did not result in accelerated weight gain (Figure 3K and 3L).

### Overexpression of LRP1 Rescues the Metabolic Phenotype and Leptin Signaling

**Figure 4:**
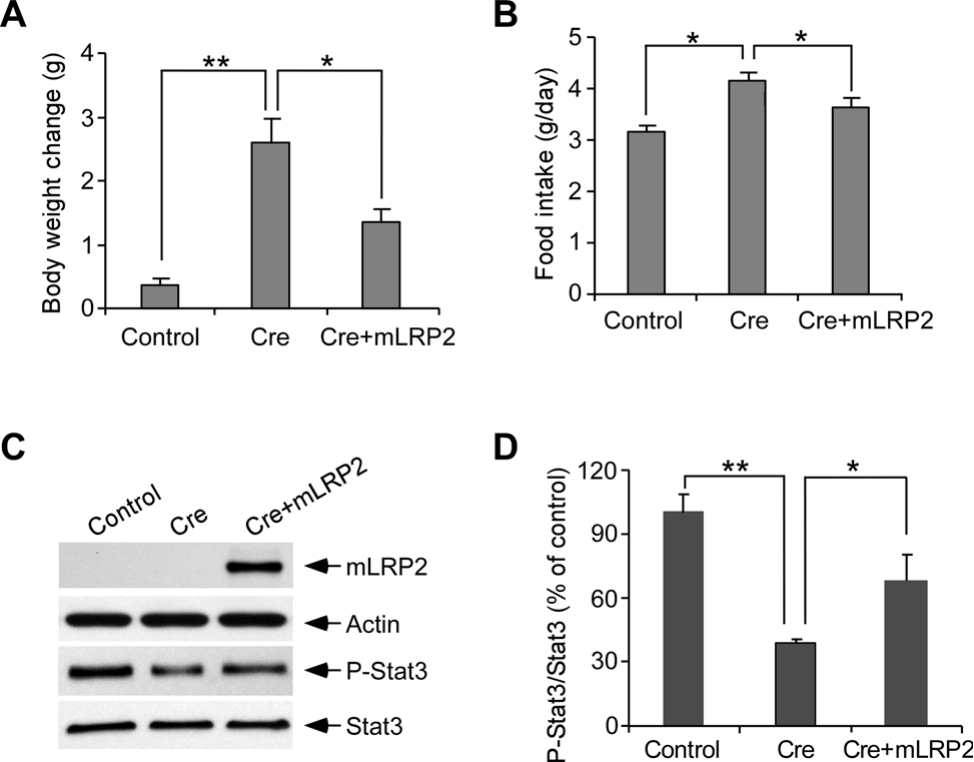
LRP1 overexpression in LRP1 hypothalamic knockdown mice rescues leptin signaling and metabolic phenotype. (A–D) *Lrp1* floxp mice were injected bilaterally in the hypothalamus with Cre lentivirus, Cre lentivirus plus mLRP2 lentivirus, or control GFP lentivirus. (A, B) Body weight (A) and food intake (B) were measured after lentivirus injection (*n* = 6, *p<0.05; **p<0.01). (C) Levels of mLRP2, P-Stat3, total Stat3, and actin in the hypothalamus were analyzed by Western blotting. (D) Densitometric analyses of Western blot samples (*n* = 4, *p<0.05; **p<0.01) indicate that LRP1 deletion in the hypothalamus significantly decreased the ratio of P-Stat3/Stat3, which was rescued by mLRP2 overexpression. Error bars are mean ± s.e.m.

Having demonstrated that deletion of LRP1 led to obesity and decreased leptin signaling, we were prompted to evaluate whether overexpression of LRP1 could rescue the obese phenotype. Co-expression of mLRP2, a functional LRP1 minireceptor [21], reduced the body weight gain and ameliorated the hyperphagia associated with hypothalamic cre overexpression in *Lrp1* floxp mice (Figure 4A and 4B). mLRP2 also partially restored leptin signaling (Figure 4C and 4D). Finally, we examine if LRP1 was also involved in the obese phenotype of ob/ob mice. LRP1 protein expression levels were significantly decreased in the hypothalamus of ob/ob mice when compared to wild type mice (Figure S11A and S11B). These results strongly support a critical role for LRP1 in leptin signaling in the hypothalamus.

Leptin binding to the long form of the leptin receptor (ObRb) stimulates the tyrosine kinase JAK2 to phosphorylate Stat3 at tyrosine residues. P-Stat3 dimers subsequently enter the nucleus and regulate transcription of target genes, such as AgRP and POMC [13]–[15]. We found that LRP1 directly binds to leptin and the leptin receptor complex and is required for leptin receptor phosphorylation and Stat3 activation. Further, LRP1 knockdown in GT1-7 cells decreased the interaction between leptin receptor and JAK2, which reduced the leptin-mediated phosphorylation of JAK2. Interestingly, LRP1 is expressed in AgRP but not in POMC neurons, and disruption of LRP1 significantly increases the expression of AgRP and NPY, indicating that LRP1 deletion impairs lepitn signaling in AgRP neurons. Taken together, our results suggest that LRP1 modulates leptin signaling likely via regulation of JAK2 activation in AgRP neurons.

It has been reported that leptin receptors are also expressed in various cortical regions and hippocampus [22]–[23], which are associated with learning and memory. In leptin receptor-deficient mice, hippocampal CA1 region exhibits impaired long-term potentiation (LTP) and long-term depression (LTD) [24], which are widely considered major cellular mechanisms underlying learning and memory. Further, leptin enhances NMDA receptor function and modulates hippocampal synaptic plasticity [25]. LRP1 is also widely expressed in the cortex and hippocampus and play essential roles in neurotransmission, and synaptic plasticity [5]–[7]. LRP1 deletion in the cortex and hippocampus region may also decrease leptin signaling and further affect synaptic function, learning, and memory.

Neuronal inactivation of LRP1 achieved by expression of the Cre transgene under control of the synapsin I promoter [26] has been previously reported [27]. Unlike the CamKII-Cre model, in which *Lrp1* disruption was restricted to the forebrain and hypothalamic areas, LRP1 deletion in the Syn-Cre model was detectable much earlier and throughout the entire CNS, first manifesting by impairment of motor function and systemic tremors about 3 wk after birth. SynI-Cre/*Lrp1* mutant mice were also hyperphagic, but in contrast to the CamKII-Cre/*Lrp1* model, they were hyperkatabolic, and the majority of Syn-Cre/*Lrp1* mice died prematurely between 6 and 9 mo of age. This severe and earlier phenotype emphasized a critical role for CNS LRP1 in motor circuitry and muscle control but effectively occluded another pivotal role for LRP1 in the CNS, i.e., the regulation of leptin signaling and metabolic homeostasis described here.

In summary, we have uncovered a new molecular pathway that regulates leptin signaling and energy homeostasis. Our results demonstrate that LRP1, a lipoprotein receptor that plays important roles in lipid metabolism, also regulates food intake and body energy homeostasis in the CNS. Because ICV administration of apoE was recently shown to suppress food intake in the rat [28], it is plausible to speculate that brain lipoproteins may also play a role in regulating food intake, likely via LRP1. Interestingly, we found that LRP1 knockout resulted in increased apoE levels in the hypothalamus (Figure S12), consistent with the fact that apoE metabolism is impaired in the LRP1 deficient mice. Therefore, LRP1 provides a critical link between peripheral and central energy metabolism and could serve as a novel therapeutic target to lessen obesity in human patients.

## Materials and Methods

### Materials

All tissue culture media and serum were from Sigma. Anti-leptin receptor antibody was purchased from Santa Cruz, anti-JAK2 antibody from Invitrogen, anti-phosphotyrosine antibody from Upstate, anti-P-Stat3 and anti-Stat3 antibodies from Cell Signaling, and anti-actin antibody from Sigma. In-house anti-LRP1 antibody has been described previously [29]–[30]. Peroxidase-labeled anti-mouse antibody and ECL system were from GE Healthcare. Carrier-free Na^125^I was purchased from Perkin Elmer Lifescience. Recombinant human insulin was from Eli Lilly and recombinant mouse leptin was from R&D Systems.

### Food Intake and Metabolic Measurements

For feeding studies, animals were singly housed for 2 wk. Food intake was measured daily for consecutive 7-d or 14-d periods. Metabolic rates were measured by indirect calorimetry (Windows Oxymax Equal Flow system, Columbus Instruments) at the Washington University Diabetes Research and Training Center (DRTC). Mice were housed individually in air-tight respiratory cages through which room air was passed at a flow rate of 0.51/min. O_2_ and CO_2_ contents of exhausted air were determined by comparison with O_2_ and CO_2_ contents of standardized sample air. VO2 and VCO2 were normalized to lean body mass [31].

For GTT studies, mice were fasted overnight and D-glucose at 2 g/kg of body weight was injected intraperitoneally. Blood glucose was monitored at 0, 15, 30, 60, 90, and 120 min after glucose injection. Results were expressed as mean blood glucose concentration from at least six animals per genotype. For ITT studies, mice were fasted for 6 h and human insulin at 1 IU/kg of body weight was injected intraperitoneally. Blood glucose levels were monitored at 0, 15, 30, 60, and 120 min after insulin injection. Results were expressed as mean percent of basal blood glucose concentration from at least seven animals per genotype.

For leptin sensitivity, 12-mo-old mice were fasted for 24 h and injected intraperitoneally with leptin (1 mg/kg body weight) or PBS as control. Hypothalamic extracts were prepared 45 min after injection and immunoblotted with P-Stat3 and Stat3 antibody.

For chronic intracerebroventricular (ICV) infusion of leptin, stereotaxic implantation of an intraventricular cannula to the third ventricle of 12-mo-old mice was performed by the Washington University Hope Center In Vivo Animal Models Core. After 1 wk, an osmotic mini-pump (Alzet minipump) was attached to the ICV catheter. The mini-pump delivered a constant infusion of leptin (50 ng/h) or artificial cerebrospinal fluid (aCSF) for 14 d. Food intake and body weight were monitored.

### Body Fat Content

MRI, a whole-body magnetic resonance analyzer for mice (Echo Medical Systems), was used to perform quantitative magnetic resonance analysis of fat-free mass and fat mass. Fat content was calculated as a percent of fat mass over the total body mass.

### Real-Time Reverse Transcriptase-Polymerase Chain Reaction (RT-PCR)

RT-PCR analysis was performed as described previously [14]. Details can be found in Text S1.

### Small Interfering RNA (siRNA)-Mediated Knockdown of LRP1 in GT1-7 Cells

ON-TARGET plus™ siRNA for LRP1 and control siRNA were purchased from Dharmacon Research. Cells were transiently transfected with 50 nM of the siRNA duplex using Lipofectamine 2000 (Invitrogen) according to the manufacturer's instructions and harvested for processing 48 h post-transfection.

### Chemical Cross-Linking and Immunoprecipitation

Experiments were performed with ^125^I-leptin cross-linked to unlabeled GT1-7 cells as described before [20]. Details can be found in Text S1.

### Western Blotting

Western blot analysis was performed as described previously [20]. Details can be found in Text S1.

### Plasma Lipid, Insulin, and Leptin Analyses

Venous blood was taken from 12-mo-old mice and centrifuged at 7,000 rpm at 4°C for 5 min to separate plasma. Plasma FFAs, TG, and cholesterol analyses were performed by the Washington University DRTC. Serum leptin and insulin levels were also measured by the DRTC using ELISA methods.

### Generation of Lentivirus and Hypothalamus Injection

The lentivirus plasmids pHR'EF-Cre-WPRE-SIN and pHR-EF-GFP-WPRE-SIN have been described previously [32]. Cre lentivirus and GFP lentivirus were produced by the Washington University Hope Center Viral Vectors Core. Eight to 10-wk-old Rosa-26 reporter mice [33] or *Lrp1* floxp mice were stereotaxically injected with lentivirus into the arcuate nucleus of the hypothalamus (4 µl, 4.6×10^8^ TU/ml) with an air pressure injector system. The injection was performed by the Washington University Hope Center In Vivo Animal Models Core.

### β-Gal Staining

Staining was performed as described previously [32]. Details can be found in Text S1.

### Immunofluorescence Staining

Staining was performed as described previously [14]. Details can be found in Text S1.

### Statistical Analysis

All data represent the average of at least triplicate samples. Error bars represent standard error of the mean. Statistical significance was determined by Student's *t* test and *p*<0.05 was considered significant.

## Supporting Information

Figure S1 ***Lrp1*** **deletion in brain and peripheral tissues in LRP1 forebrain knockout mice.** (A–D) LRP1 expression levels were compared between LRP1-KO (*Lrp1^flox+/+^/Cre^+/^* ^−^, LRP1 knockout) and WT (*Lrp1^flox+/+^/Cre* ^−*/*−^, *Lrp1* floxP littermate control) mice at 3, 6, 9, and 12 mo of age by Western blotting. Densitometric analysis of Western blots from multiple samples (*n* = 4) indicated that LRP1 expression was significantly reduced in an age-dependent manner in the cortex (A), hypothalamus (B), and hippocampus (C), but not in the cerebellum (D) of LRP1-KO mice. *p<0.05; **p<0.01; N.S., not significant. For Panels A–D, data are presented as mean ± s.e.m. (E) LRP1 expression levels in selected peripheral tissues were compared between LRP1-KO and WT mice at 12 mo of age via Western blotting. LRP1 expression levels were not significantly altered in white adipose tissue, brown adipose tissue, pancreas, lung, spleen, liver, heart, intestine, muscle, or kidney of LRP1-KO mice.
(1.34 MB TIF)

Figure S2 **Neuronal deletion of** ***Lrp1*** **in adult brain leads to decreased leptin signaling and obesity.** (A) Body weights of female LRP1-KO and WT mice were determined at indicated ages (*n* = 10). *p<0.05; **p<0.01. (B) Neuronal deletion of *Lrp1* in adult brain did not change snout-anus length. Snout-anus length of 12-mo-old LRP1-KO and WT mice were measured (*n* = 5, N.S., not significant). (C–D) Hypothalamic P-Stat3 levels were decreased in neuronal LRP1-KO mice. (C) Levels of P-Stat3 and total Stat3 in the hypothalamus of LRP1-KO and WT mice at 9 mo of age were evaluated by Western blotting. (D) Densitometric quantification of P-Stat3 and total Stat3 levels was performed as described in Materials and Methods (*n* = 4, *p<0.05). Data are presented as mean ± s.e.m.
(0.25 MB TIF)

Figure S3 **LRP1 is expressed in AgRP neurons but not in POMC neurons of the mouse hypothalamus.** Double immunofluorescence staining was performed using an anti-LRP1 antibody (detected with Alexa 568, red) together with either an anti-AgRP antibody or anti-MSH antibody (detected with Alexa 488, green). Representative staining images in mouse hypothalamus are shown. Note that LRP1 colocolized with AgRP but not with POMC stained neurons.
(1.44 MB TIF)

Figure S4 **LRP1 knockdown in GT1-7 cells decreases Stat3 and ERK signaling.** (A) GT1-7 cells were transiently transfected with control siRNA or LRP1-specific siRNA for 48 h. Levels of LRP1, P-Stat3, and total Stat3 were analyzed by Western blotting. (B–C) LRP1 knockdown in GT1-7 cells reduced ERK phosphorylation. (B) GT1-7 cells were transiently transfected with HA-ObRb and control siRNA or LRP1-specific siRNAs for 48 h, serum-starved overnight, and then treated with 50 nM leptin for 30 min. Levels of P-ERK1/2 and total ERK1/2 were analyzed by Western blotting. (C) Densitometric analysis of blots (*n* = 4, *p<0.05) indicated that LRP1 knockdown significantly reduced the ratio of P-ERK/ERK. Data are shown as mean ± s.e.m.
(0.35 MB TIF)

Figure S5 **LRP1 knockdown in GT1-7 cells decreases Stat3 phosphorylation.** (A) GT1-7 cells were transiently transfected with control siRNA, LRP1-specific siRNA, and/or HA-ObR cDNA for 48 h, serum-starved overnight, and then treated with 50 nM leptin for 30 min. Levels of HA-ObR, P-Stat3, and total Stat3 were analyzed by Western blotting. (B) Densitometric analysis of blots (*n* = 4, **p<0.01, *p<0.05) indicated that ObR overexpression alone increased the ratio of P-Stat3/Stat3 and that LRP1 knockdown markedly reduced leptin-mediated increase of P-Stat3/Stat3. Data are shown as mean ± s.e.m.
(0.31 MB TIF)

Figure S6 **LRP1 interacts with leptin and leptin receptor complex**. (A) Levels of ObR and actin in the hypothalamus of wild-type and db/db mice (Jackson lab) at 12 wk of age were evaluated by Western blotting. (B) Cellular extracts were prepared from GT1-7 cells and immunoprecipitated with either a control antibody or an anti-JAK2 antibody followed by immunoblotting with an anti-ObR or anti-JAK2 antibody. Extracts were also directly immunoblotted with the ObR antibody and JAK2 antibody. (C) Cellular extracts prepared from GT1-7 cells were immunoprecipitated with either a control antibody or an anti-ObR antibody, followed by immunoblotting with an anti-LRP1 or anti-ObR antibody. Extracts were also directly immunoblotted with the ObR antibody and LRP1 antibody. (D) Extracts were prepared from GT1-7 cells and immunoprecipitated with either an anti-JAK2 antibody or an anti-JAK2 antibody with specific blocking peptide, followed by immunoblotting with an anti-ObR or anti-JAK2 antibody. Extracts were also directly immunoblotted with the ObR antibody and JAK2 antibody. (E) Extracts were prepared from GT1-7 cells and immunoprecipitated with either an anti-ObR antibody or an anti-ObR antibody with specific blocking peptide, followed by immunoblotting with an anti-LRP1 or anti-ObR antibody. Extracts were also directly immunoblotted with the ObR antibody and LRP1 antibody. (F) Densitometric quantification of immunoreactive bands from Figure 2L was performed as described in Materials and Methods (*n* = 4, N.S., not significant).
(0.71 MB TIF)

Figure S7 **LRP1 knockdown in GT1-7 cells decreases JAK2 phosphorylation.** (A) GT1-7 cells were transiently transfected with HA-ObRb for 48 h, serum-starved overnight, and then treated with 50 nM leptin or vehicle control for 30 min. Extracts were prepared from GT1-7 cells and immunoprecipitated with an anti-HA antibody followed by immunoblotting with an anti-JAK2 antibody. Extracts were also directly immunoblotted with the HA antibody, JAK2 antibody, and Actin antibody. (B) GT1-7 cells were transiently transfected with control siRNA, LRP1-specific siRNA, and/or HA-ObR for 48 h, serum-starved overnight, and then treated with 50 nM leptin for 30 min. Levels of P-JAK2 and total JAK2 were analyzed by Western blotting. (C) Densitometric analysis of blots (*n* = 4, **p<0.01, *p<0.05) indicated that, similar to Stat3, ObR overexpression alone increased the ratio of P-JAK2/JAK2 and that LRP1 knockdown reduced leptin-mediated increase of P-JAK2/JAK2. Data are shown as mean ± s.e.m.
(0.37 MB TIF)

Figure S8 **LRP1 is directly associated with the leptin/leptin receptor complex.** (A) GT1-7 cells were transiently transfected with control or HA-ObR for 48 h. Ligand binding was performed by incubating GT1-7 cells with ^125^I-leptin for 1 h at 4°C. Chemical crosslinking was carried out, followed by immunoprecipitation with control antibody, anti-LRP1 antibody, anti-leptin antibody, anti-HA antibody, and immunoprecipitates were analyzed on SDS-PAGE. (B) Densitometric quantification of immunoreactive bands from (A) was performed as described in Materials and Methods (*n* = 4, N.S., not significant).
(0.39 MB TIF)

Figure S9 **Cre lentivirus injection into the hypothalamus of the** ***Lrp1*** **floxp mice leads to LRP1 deletion.** *Lrp1* floxp mice (*Lrp1^flox+/+^/Cre* ^−*/*−^) mice were bilaterally injected into ARC of the hypothalamus with either Cre lentivirus or control GFP lentivirus. Fourteen days after lentivirus injection, immunofluorescence staining was performed using an anti-LRP1 antibody (detected with Alexa 488, green) and a representative staining in ARC of hypothalamus is shown.
(0.81 MB TIF)

Figure S10 **Cre lentivirus injection into** ***Lrp1*** **floxp mice leads to decreased leptin sensitivity.** *Lrp1* floxp mice were bilaterally injected with either Cre lentivirus or control GFP lentivirus. (A) Fourteen days after lentivirus injection, *Lrp1* floxp mice were injected intraperitoneally with leptin (1 mg/kg body weight) or PBS as control. Hypothalamic extracts were prepared 45 min after injection. Levels of P-Stat3 and total Stat3 were analyzed by Western blotting. (B) Densitometric quantification of P-Stat3 and total Stat3 levels was performed as described in Materials and Methods (*n* = 4, *p<0.05; **p<0.01).
(0.18 MB TIF)

Figure S11 **LRP1 expression levels are significantly decreased in ob/ob mice.** (A) LRP1 expression levels in the hypothalamus were compared between WT and ob/ob mice at 4 mo of age by Western blotting. LRP1 expression levels were significantly decreased in the hypothalamus of ob/ob mice. (B) Densitometric analysis of Western blot samples (*n* = 4, *p<0.05).
(0.11 MB TIF)

Figure S12 **LRP1 deletion increases apoE levels in the hypothalamus.** ApoE levels were measured in hypothalamic lysates of 13-mo-old WT and LRP1-KO mice (*n* = 5), normalized against total protein, and plotted as a percentage of WT controls. *p<0.05.
(0.05 MB TIF)

Text S1 **Supplementary experimental procedures.**
(0.04 MB DOC)

## Abbreviations

AD: Alzheimer's disease
AgRP: agouti-related protein
CNS: central nervous system
ERK: extracellular signal-regulated kinase
FFA: free fatty acid
GTT: glucose tolerance test
ICV: introcerebroventricular
ITT: insulin tolerance test
JAK2: janus kinase 2
LDLR: LDL receptor
LTD: long-term depression
LTP: long-term potentiation
LRP1: lipoprotein receptor-related protein 1
LRP1-KO: *Lrp1* forebrain knockout mice
NPY: neuropeptide Y
P-Stat3: phospho-Stat3
TG: triglyceride
