# Correction: Lipoprotein Receptor LRP1 Regulates Leptin Signaling and Energy Homeostasis in the Adult Central Nervous System

**DOI:** 10.1371/journal.pbio.3000310

**Published:** 2019-06-04

**Authors:** Qiang Liu, Juan Zhang, Celina Zerbinatti, Yan Zhan, Benedict J. Kolber, Joachim Herz, Louis J. Muglia, Guojun Bu

The authors would like to clarify two issues recently raised by the *PLOS Biology* editors.

The image included in the original publication for S1E Fig, right panel, LRP1 blot, is incorrect. This image was erroneously constructed during the submission process to *PLOS Biology*. Additionally, several images of gels were unintentionally altered during processing that resulted in discontinuous bands (including Panel A Actin, Panel B LRP1 and Actin, Panel C Actin, Panel D LRP1 and Actin, and Panel E GAPDH of right panel). The authors have provided a correct figure that resolves these issues.

## Supporting information

S1 Fig *Lrp1* deletion in brain and peripheral tissues in LRP1 forebrain knockout mice. (A–D) LRP1 expression levels were compared between LRP1-KO (*Lrp1*^*flox+/+*^*/Cre*^*+/*−^, LRP1 knockout) and WT (*Lrp1*^*flox+/+*^*/Cre*^−*/*−^, *Lrp1* floxP littermate control) mice at 3, 6, 9, and 12 mo of age by Western blotting. Densitometric analysis of Western blots from multiple samples (*n* = 4) indicated that LRP1 expression was significantly reduced in an age-dependent manner in the cortex (A), hypothalamus (B), and hippocampus (C), but not in the cerebellum (D) of LRP1-KO mice. *p<0.05; **p<0.01; N.S., not significant. For Panels A–D, data are presented as mean ± s.e.m. (E) LRP1 expression levels in selected peripheral tissues were compared between LRP1-KO and WT mice at 12 mo of age via Western blotting. LRP1 expression levels were not significantly altered in white adipose tissue, brown adipose tissue, pancreas, lung, spleen, liver, heart, intestine, muscle, or kidney of LRP1-KO mice.
(TIF)
