# Expression of Concern: Lipoprotein Receptor LRP1 Regulates Leptin Signaling and Energy Homeostasis in the Adult Central Nervous System

**DOI:** 10.1371/journal.pbio.3003528

**Published:** 2025-12-01

**Authors:** The PLOS Biology Editors

Following the publication and subsequent correction of this article [1,2], concerns were raised regarding results presented in Figs 2, S4, S6, and S8. Specifically,

- The Fig 2J Direct WB LRP1 panel appears similar to the Fig S4A LRP1 panel when flipped and rotated, despite being used to represent different conditions.
- The Fig 2J Direct WB Actin panel appears similar to the Fig S4A Actin panel, despite being used to represent different conditions.
- The Fig S6C Direct WB LRP1 panel appears similar to the Fig S6D Direct WB JAK2 panel.

The authors stated that errors were made during the preparation of Figs S4 and S6, and submitted the available blots underlying Figs 2, S1, S4, S6, and S8 for editorial review (S1 File). Editorial assessment of the underlying data confirmed a figure preparation error affecting Fig S6, but similarities between images provided for Figs 2J and S4A raised further concerns about the reliability of the results presented in these figures. An updated Fig S6 presenting the correct Direct WB JAK2 results is provided with this notice.

In addition, during the assessment of the underlying data provided for Fig S8A, the Editors noted that the underlying blots provided for the right Fig S8A panel do not appear to match the published figure, calling into question the reliability of the associated quantified results presented in Fig S8B. Author QL commented that the underlying blots provided in support of the Fig S8A original α-HA and α-LRP1 image data could not be retrieved and that the right panel provided as underlying blots originates from an independently repeated experiment instead. In the absence of the uncropped blots underlying all the original results PLOS remains concerned about the reliability of the Fig S8 results.

In light of the concerns raised, the *PLOS Biology* Editors issue this Expression of Concern to notify readers, to relay the blots provided by the corresponding author, and to inform readers that the Fig 2, Fig S4, and Fig S8 results should be interpreted with caution.

## Supporting information

S1 File Available blots underlying results presented in Figs 2, S1, S4, S6, and S8. (ZIP)

Fig S6 LRP1 interacts with leptin and leptin receptor complex. (A) Levels of ObR and actin in the hypothalamus of wild-type and db/db mice (Jackson lab) at 12 wk of age were evaluated by Western blotting. (B) Cellular extracts were prepared from GT1–7 cells and immunoprecipitated with either a control antibody or an anti-JAK2 antibody followed by immunoblotting with an anti-ObR or anti-JAK2 antibody. Extracts were also directly immunoblotted with the ObR antibody and JAK2 antibody. (C) Cellular extracts prepared from GT1–7 cells were immunoprecipitated with either a control antibody or an anti-ObR antibody, followed by immunoblotting with an anti-LRP1 or anti-ObR antibody. Extracts were also directly immunoblotted with the ObR antibody and LRP1 antibody. (D) Extracts were prepared from GT1–7 cells and immunoprecipitated with either an anti-JAK2 antibody or an anti-JAK2 antibody with specific blocking peptide, followed by immunoblotting with an anti-ObR or anti-JAK2 antibody. Extracts were also directly immunoblotted with the ObR antibody and JAK2 antibody. (E) Extracts were prepared from GT1–7 cells and immunoprecipitated with either an anti-ObR antibody or an anti-ObR antibody with specific blocking peptide, followed by immunoblotting with an anti-LRP1 or anti-ObR antibody. Extracts were also directly immunoblotted with the ObR antibody and LRP1 antibody. (F) Densitometric quantification of immunoreactive bands from Figure 2L was performed as described in Materials and Methods (*n* = 4, N.S., not significant).
(TIF)
