# Supplementary figures and images for: Lipoprotein Receptor LRP1 Regulates Leptin Signaling and Energy Homeostasis in the Adult Central Nervous System
Source: PLoS Biol. 2011 Jan 11;9(1):e1000575. doi: 10.1371/journal.pbio.1000575 (PMC3019112; doi:10.1371/journal.pbio.1000575)

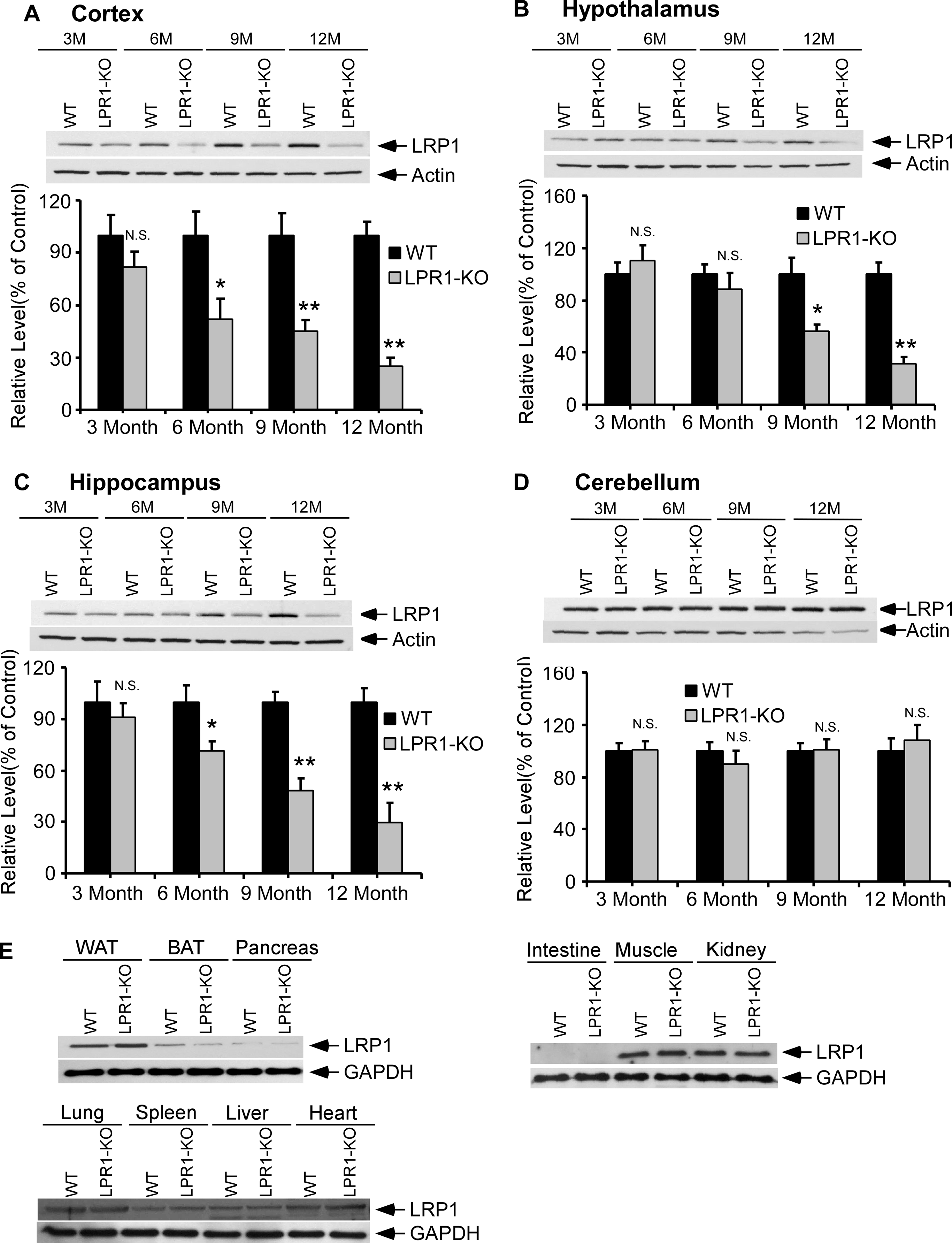

Supplement: Figure S1 — Lrp1 deletion in brain and peripheral tissues in LRP1 forebrain knockout mice. (A–D) LRP1 expression levels were compared between LRP1-KO (Lrp1flox+/+/Cre+/ −, LRP1 knockout) and WT (Lrp1flox+/+/Cre −/−, Lrp1 floxP littermate control) mice at 3, 6, 9, and 12 mo of age by Western blotting. Densitometric analysis of Western blots from multiple samples (n = 4) indicated that LRP1 expression was significantly reduced in an age-dependent manner in the cortex (A), hypothalamus (B), and hippocampus (C), but not in the cerebellum (D) of LRP1-KO mice. *p<0.05; **p<0.01; N.S., not significant. For Panels A–D, data are presented as mean ± s.e.m. (E) LRP1 expression levels in selected peripheral tissues were compared between LRP1-KO and WT mice at 12 mo of age via Western blotting. LRP1 expression levels were not significantly altered in white adipose tissue, brown adipose tissue, pancreas, lung, spleen, liver, heart, intestine, muscle, or kidney of LRP1-KO mice. (1.34 MB TIF) [file pbio.1000575.s001.tif]

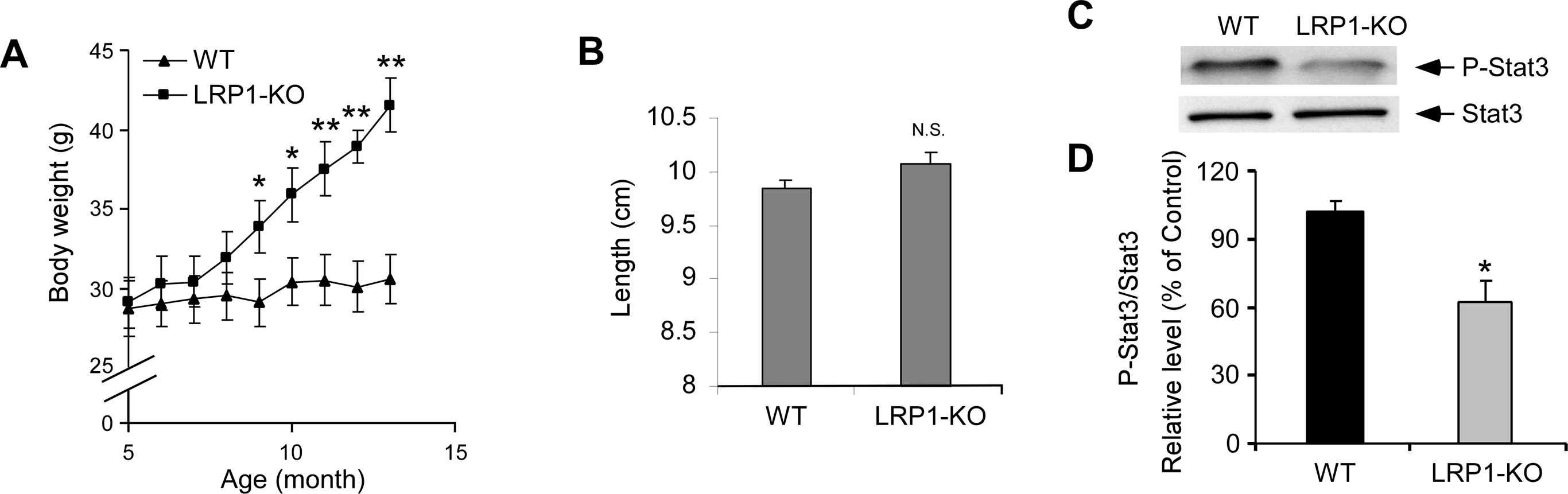

Supplement: Figure S2 — Neuronal deletion of Lrp1 in adult brain leads to decreased leptin signaling and obesity. (A) Body weights of female LRP1-KO and WT mice were determined at indicated ages (n = 10). *p<0.05; **p<0.01. (B) Neuronal deletion of Lrp1 in adult brain did not change snout-anus length. Snout-anus length of 12-mo-old LRP1-KO and WT mice were measured (n = 5, N.S., not significant). (C–D) Hypothalamic P-Stat3 levels were decreased in neuronal LRP1-KO mice. (C) Levels of P-Stat3 and total Stat3 in the hypothalamus of LRP1-KO and WT mice at 9 mo of age were evaluated by Western blotting. (D) Densitometric quantification of P-Stat3 and total Stat3 levels was performed as described in Materials and Methods (n = 4, *p<0.05). Data are presented as mean ± s.e.m. (0.25 MB TIF) [file pbio.1000575.s002.tif]

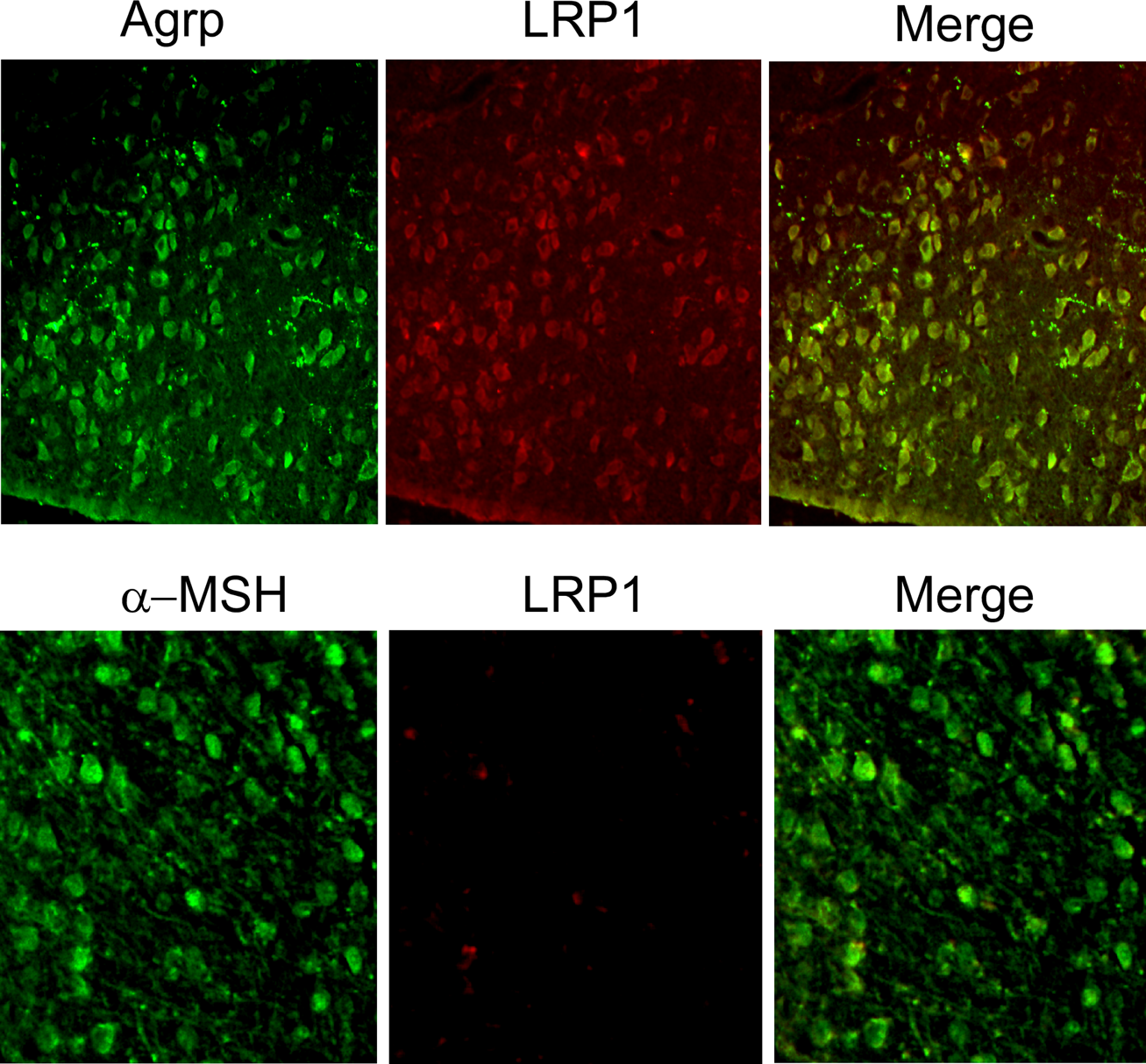

Supplement: Figure S3 — LRP1 is expressed in AgRP neurons but not in POMC neurons of the mouse hypothalamus. Double immunofluorescence staining was performed using an anti-LRP1 antibody (detected with Alexa 568, red) together with either an anti-AgRP antibody or anti-MSH antibody (detected with Alexa 488, green). Representative staining images in mouse hypothalamus are shown. Note that LRP1 colocolized with AgRP but not with POMC stained neurons. (1.44 MB TIF) [file pbio.1000575.s003.tif]

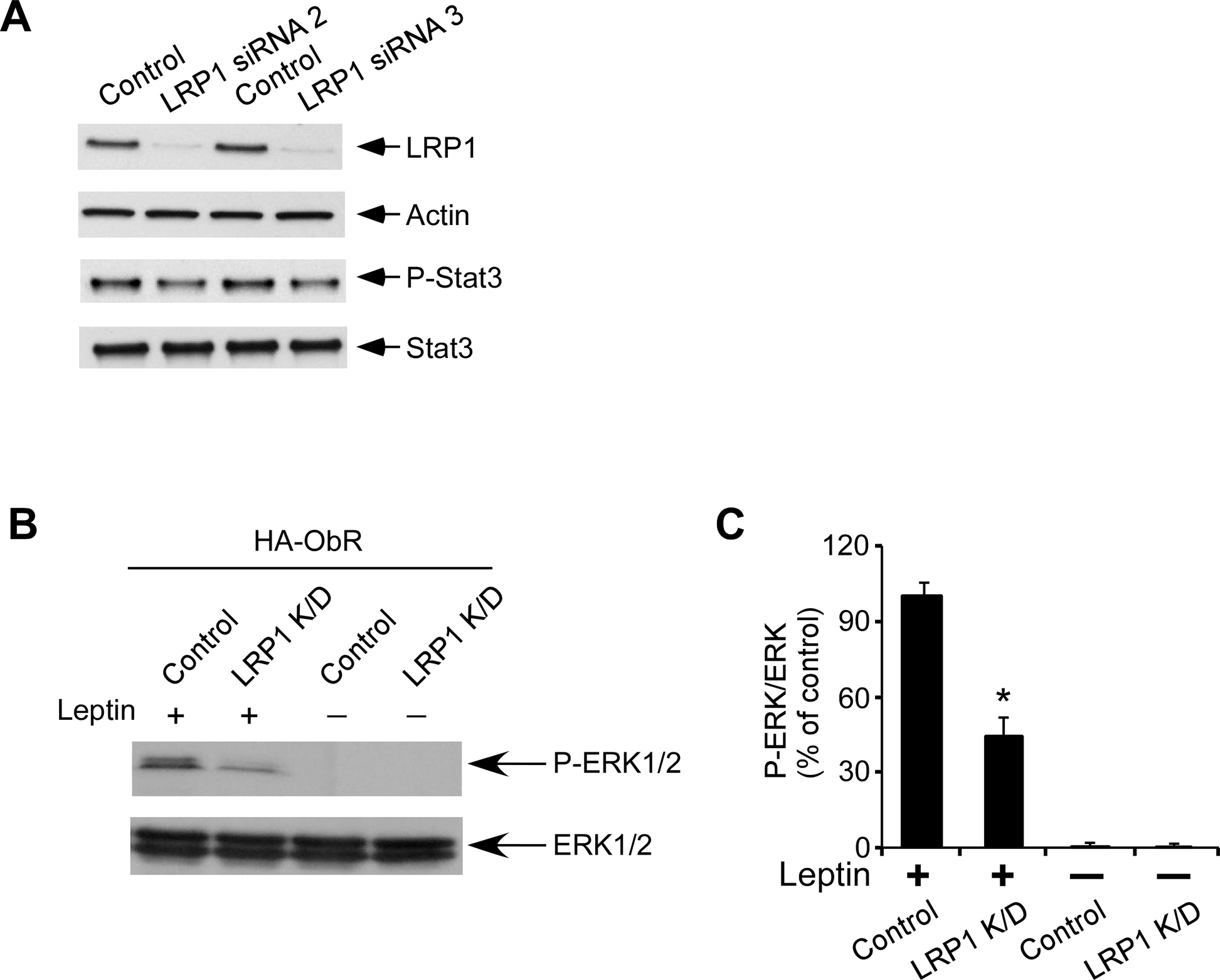

Supplement: Figure S4 — LRP1 knockdown in GT1-7 cells decreases Stat3 and ERK signaling. (A) GT1-7 cells were transiently transfected with control siRNA or LRP1-specific siRNA for 48 h. Levels of LRP1, P-Stat3, and total Stat3 were analyzed by Western blotting. (B–C) LRP1 knockdown in GT1-7 cells reduced ERK phosphorylation. (B) GT1-7 cells were transiently transfected with HA-ObRb and control siRNA or LRP1-specific siRNAs for 48 h, serum-starved overnight, and then treated with 50 nM leptin for 30 min. Levels of P-ERK1/2 and total ERK1/2 were analyzed by Western blotting. (C) Densitometric analysis of blots (n = 4, *p<0.05) indicated that LRP1 knockdown significantly reduced the ratio of P-ERK/ERK. Data are shown as mean ± s.e.m. (0.35 MB TIF) [file pbio.1000575.s004.tif]

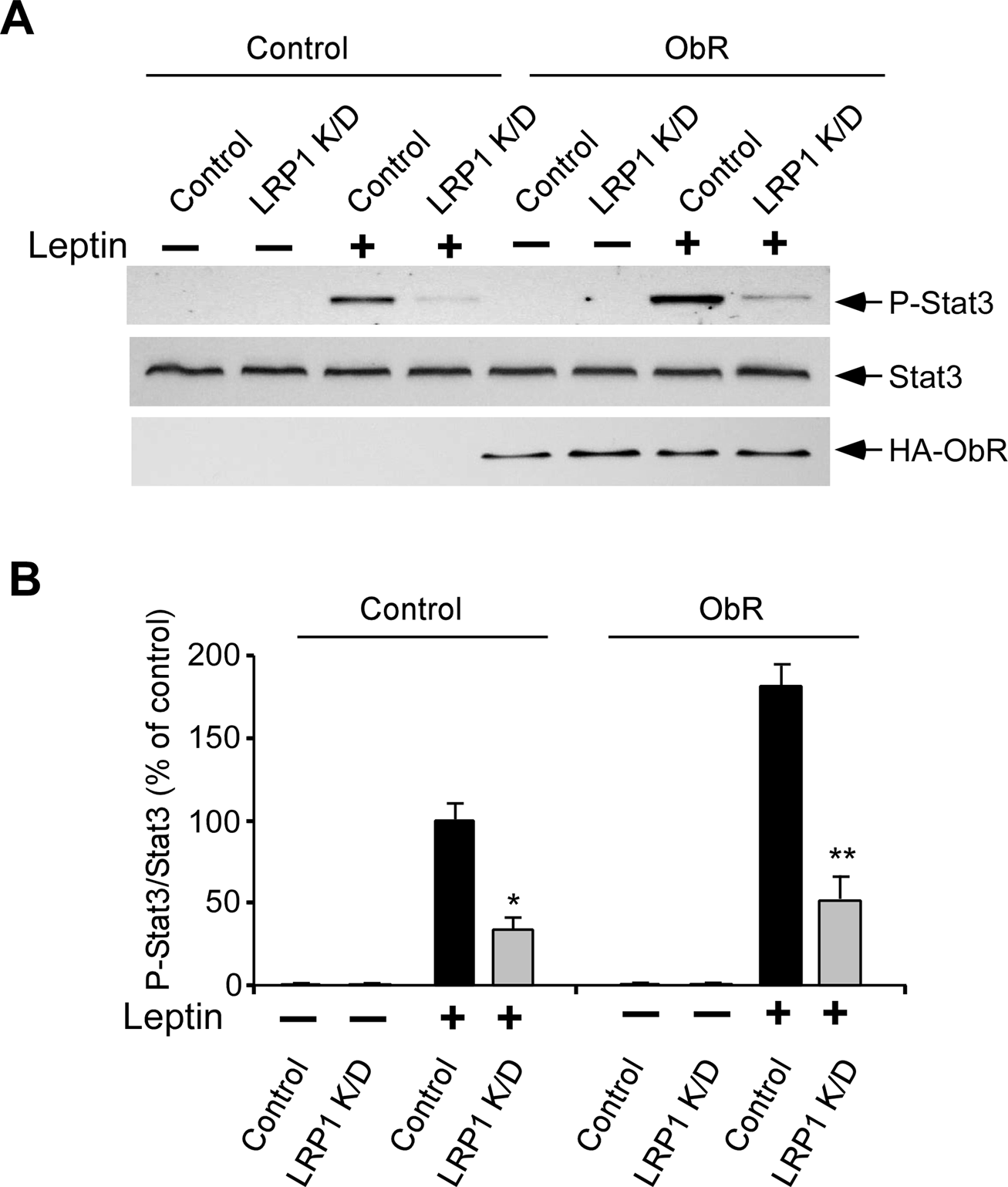

Supplement: Figure S5 — LRP1 knockdown in GT1-7 cells decreases Stat3 phosphorylation. (A) GT1-7 cells were transiently transfected with control siRNA, LRP1-specific siRNA, and/or HA-ObR cDNA for 48 h, serum-starved overnight, and then treated with 50 nM leptin for 30 min. Levels of HA-ObR, P-Stat3, and total Stat3 were analyzed by Western blotting. (B) Densitometric analysis of blots (n = 4, **p<0.01, *p<0.05) indicated that ObR overexpression alone increased the ratio of P-Stat3/Stat3 and that LRP1 knockdown markedly reduced leptin-mediated increase of P-Stat3/Stat3. Data are shown as mean ± s.e.m. (0.31 MB TIF) [file pbio.1000575.s005.tif]

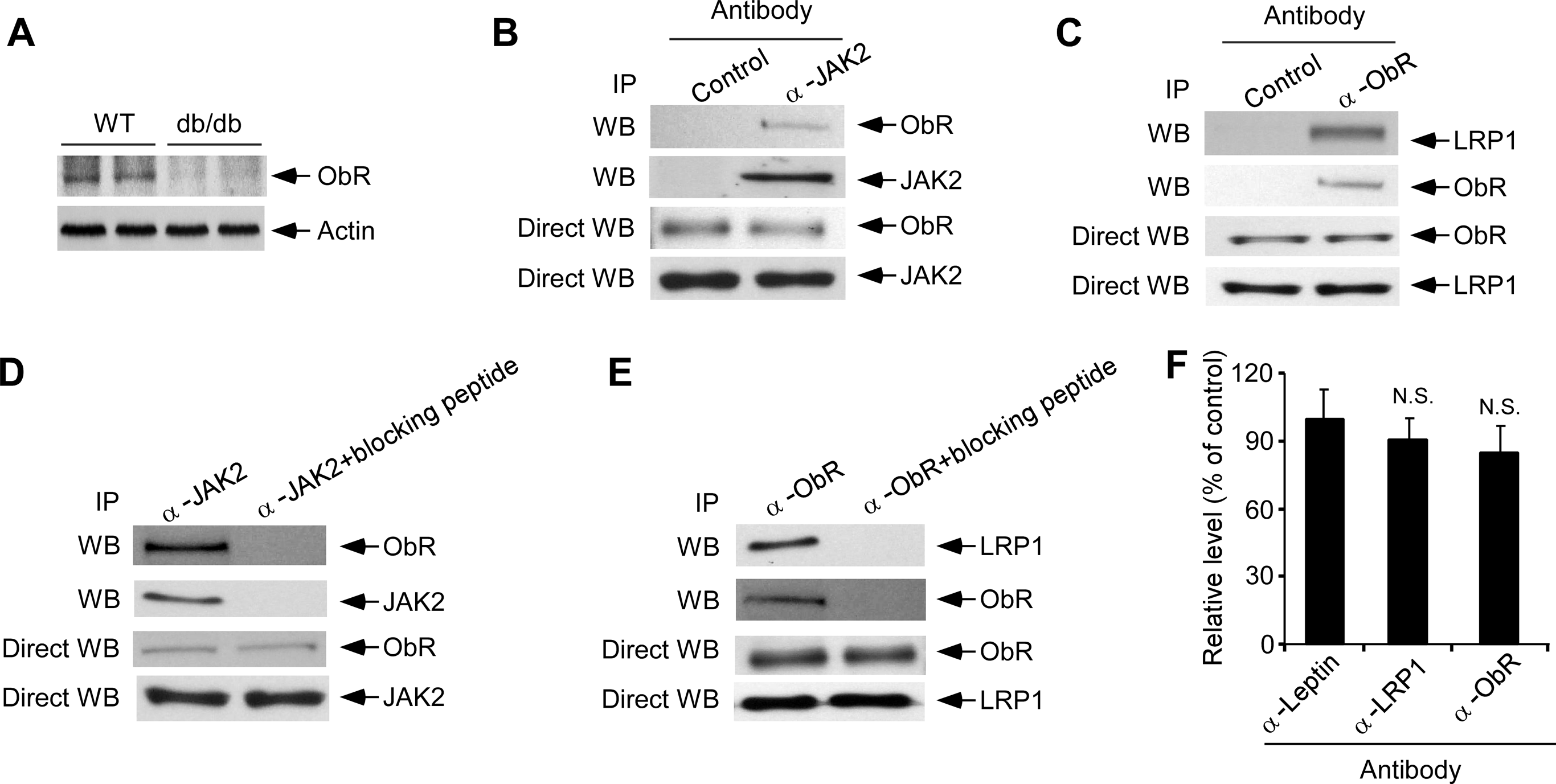

Supplement: Figure S6 — LRP1 interacts with leptin and leptin receptor complex. (A) Levels of ObR and actin in the hypothalamus of wild-type and db/db mice (Jackson lab) at 12 wk of age were evaluated by Western blotting. (B) Cellular extracts were prepared from GT1-7 cells and immunoprecipitated with either a control antibody or an anti-JAK2 antibody followed by immunoblotting with an anti-ObR or anti-JAK2 antibody. Extracts were also directly immunoblotted with the ObR antibody and JAK2 antibody. (C) Cellular extracts prepared from GT1-7 cells were immunoprecipitated with either a control antibody or an anti-ObR antibody, followed by immunoblotting with an anti-LRP1 or anti-ObR antibody. Extracts were also directly immunoblotted with the ObR antibody and LRP1 antibody. (D) Extracts were prepared from GT1-7 cells and immunoprecipitated with either an anti-JAK2 antibody or an anti-JAK2 antibody with specific blocking peptide, followed by immunoblotting with an anti-ObR or anti-JAK2 antibody. Extracts were also directly immunoblotted with the ObR antibody and JAK2 antibody. (E) Extracts were prepared from GT1-7 cells and immunoprecipitated with either an anti-ObR antibody or an anti-ObR antibody with specific blocking peptide, followed by immunoblotting with an anti-LRP1 or anti-ObR antibody. Extracts were also directly immunoblotted with the ObR antibody and LRP1 antibody. (F) Densitometric quantification of immunoreactive bands from Figure 2L was performed as described in Materials and Methods (n = 4, N.S., not significant). (0.71 MB TIF) [file pbio.1000575.s006.tif]

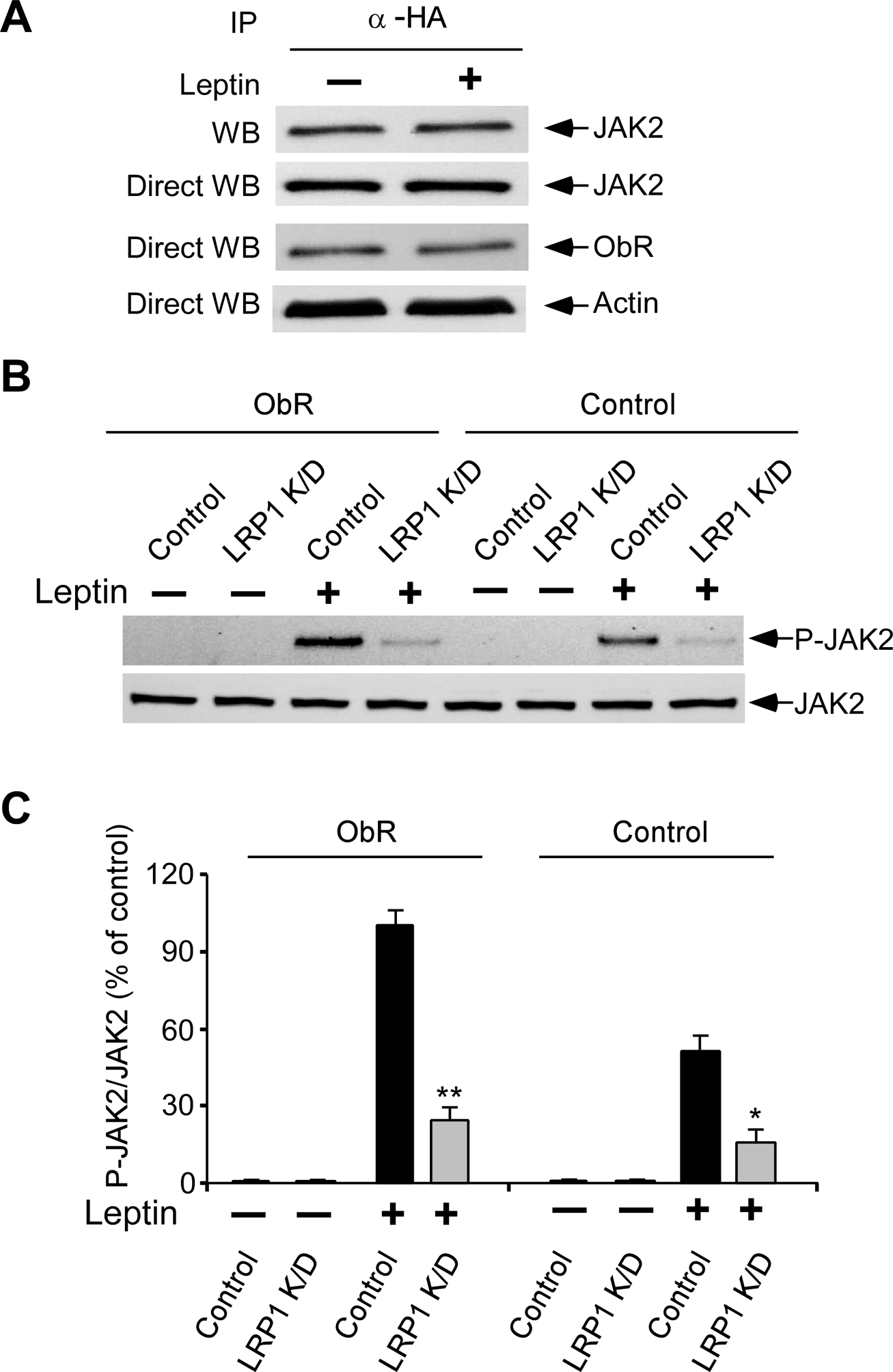

Supplement: Figure S7 — LRP1 knockdown in GT1-7 cells decreases JAK2 phosphorylation. (A) GT1-7 cells were transiently transfected with HA-ObRb for 48 h, serum-starved overnight, and then treated with 50 nM leptin or vehicle control for 30 min. Extracts were prepared from GT1-7 cells and immunoprecipitated with an anti-HA antibody followed by immunoblotting with an anti-JAK2 antibody. Extracts were also directly immunoblotted with the HA antibody, JAK2 antibody, and Actin antibody. (B) GT1-7 cells were transiently transfected with control siRNA, LRP1-specific siRNA, and/or HA-ObR for 48 h, serum-starved overnight, and then treated with 50 nM leptin for 30 min. Levels of P-JAK2 and total JAK2 were analyzed by Western blotting. (C) Densitometric analysis of blots (n = 4, **p<0.01, *p<0.05) indicated that, similar to Stat3, ObR overexpression alone increased the ratio of P-JAK2/JAK2 and that LRP1 knockdown reduced leptin-mediated increase of P-JAK2/JAK2. Data are shown as mean ± s.e.m. (0.37 MB TIF) [file pbio.1000575.s007.tif]

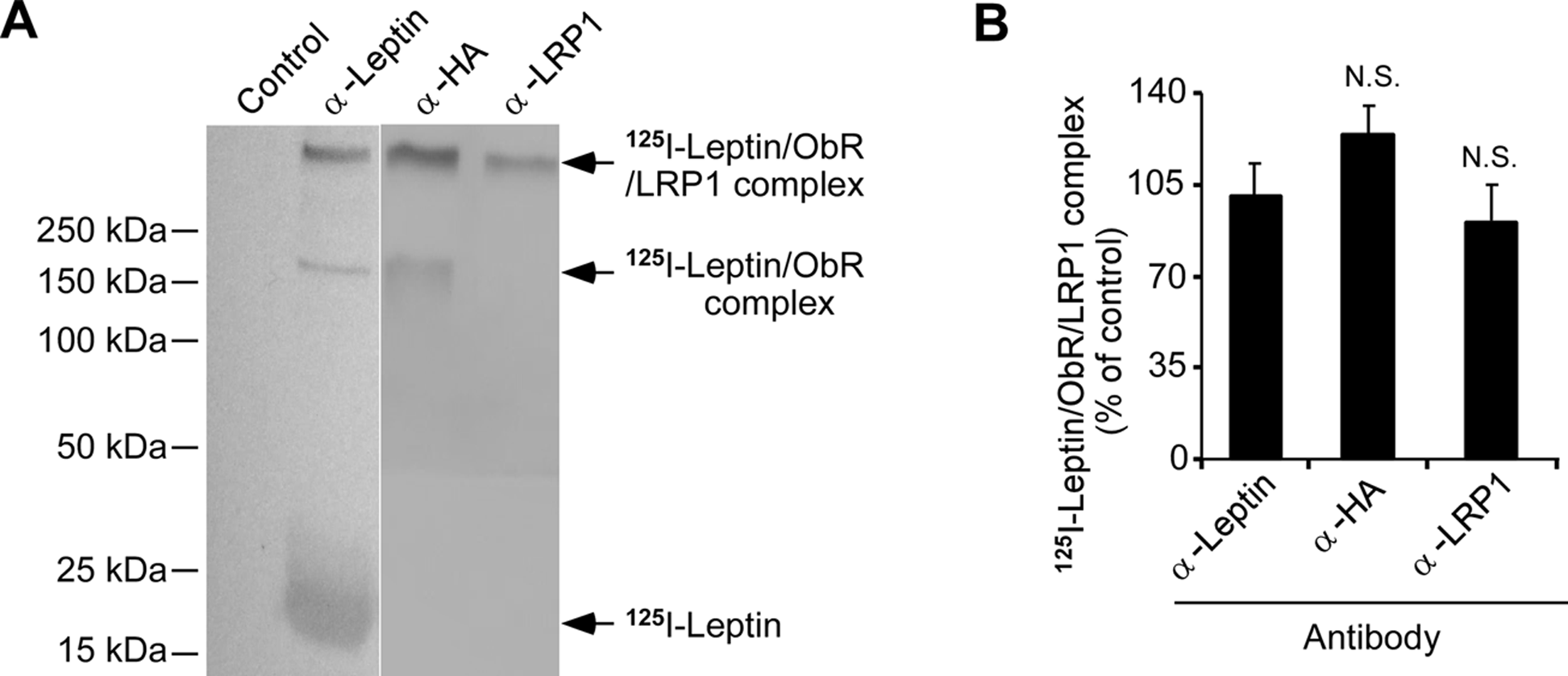

Supplement: Figure S8 — LRP1 is directly associated with the leptin/leptin receptor complex. (A) GT1-7 cells were transiently transfected with control or HA-ObR for 48 h. Ligand binding was performed by incubating GT1-7 cells with 125I-leptin for 1 h at 4°C. Chemical crosslinking was carried out, followed by immunoprecipitation with control antibody, anti-LRP1 antibody, anti-leptin antibody, anti-HA antibody, and immunoprecipitates were analyzed on SDS-PAGE. (B) Densitometric quantification of immunoreactive bands from (A) was performed as described in Materials and Methods (n = 4, N.S., not significant). (0.39 MB TIF) [file pbio.1000575.s008.tif]

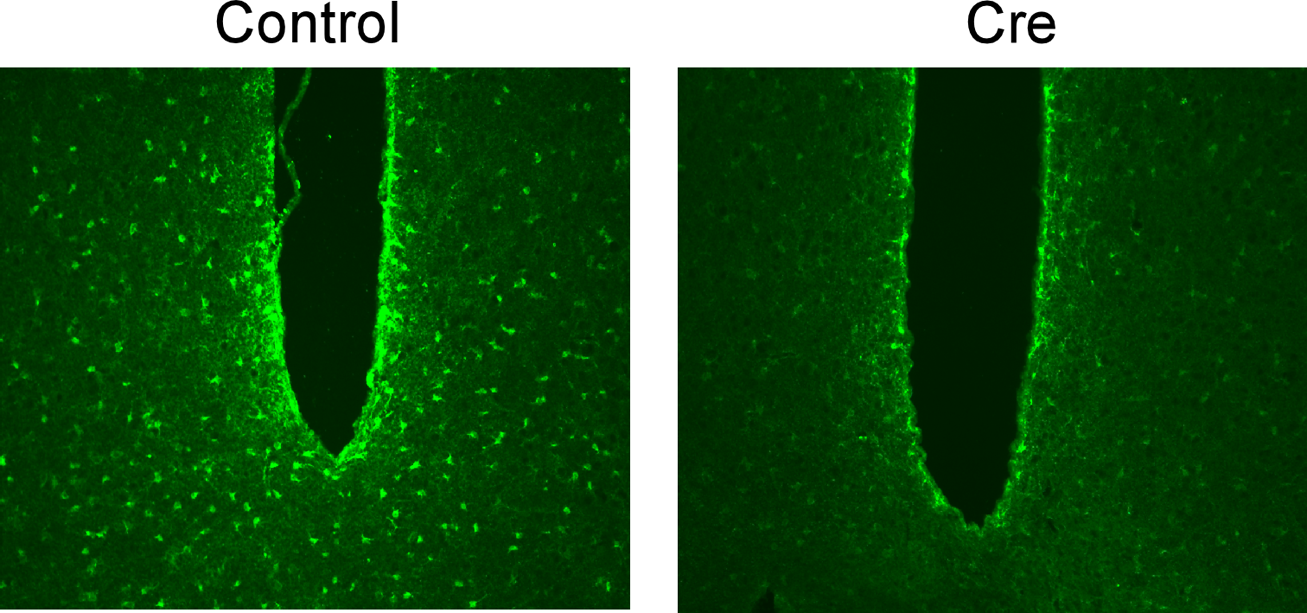

Supplement: Figure S9 — Cre lentivirus injection into the hypothalamus of the Lrp1 floxp mice leads to LRP1 deletion. Lrp1 floxp mice (Lrp1flox+/+/Cre −/−) mice were bilaterally injected into ARC of the hypothalamus with either Cre lentivirus or control GFP lentivirus. Fourteen days after lentivirus injection, immunofluorescence staining was performed using an anti-LRP1 antibody (detected with Alexa 488, green) and a representative staining in ARC of hypothalamus is shown. (0.81 MB TIF) [file pbio.1000575.s009.tif]

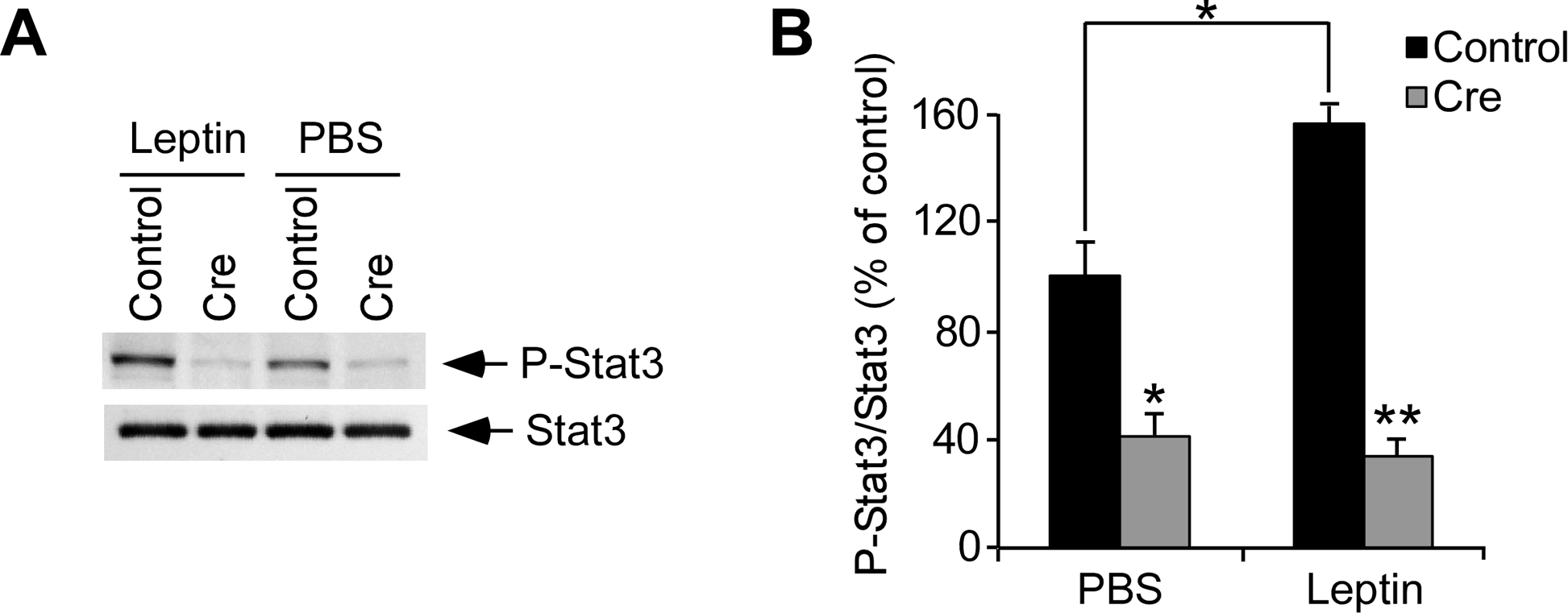

Supplement: Figure S10 — Cre lentivirus injection into Lrp1 floxp mice leads to decreased leptin sensitivity. Lrp1 floxp mice were bilaterally injected with either Cre lentivirus or control GFP lentivirus. (A) Fourteen days after lentivirus injection, Lrp1 floxp mice were injected intraperitoneally with leptin (1 mg/kg body weight) or PBS as control. Hypothalamic extracts were prepared 45 min after injection. Levels of P-Stat3 and total Stat3 were analyzed by Western blotting. (B) Densitometric quantification of P-Stat3 and total Stat3 levels was performed as described in Materials and Methods (n = 4, *p<0.05; **p<0.01). (0.18 MB TIF) [file pbio.1000575.s010.tif]

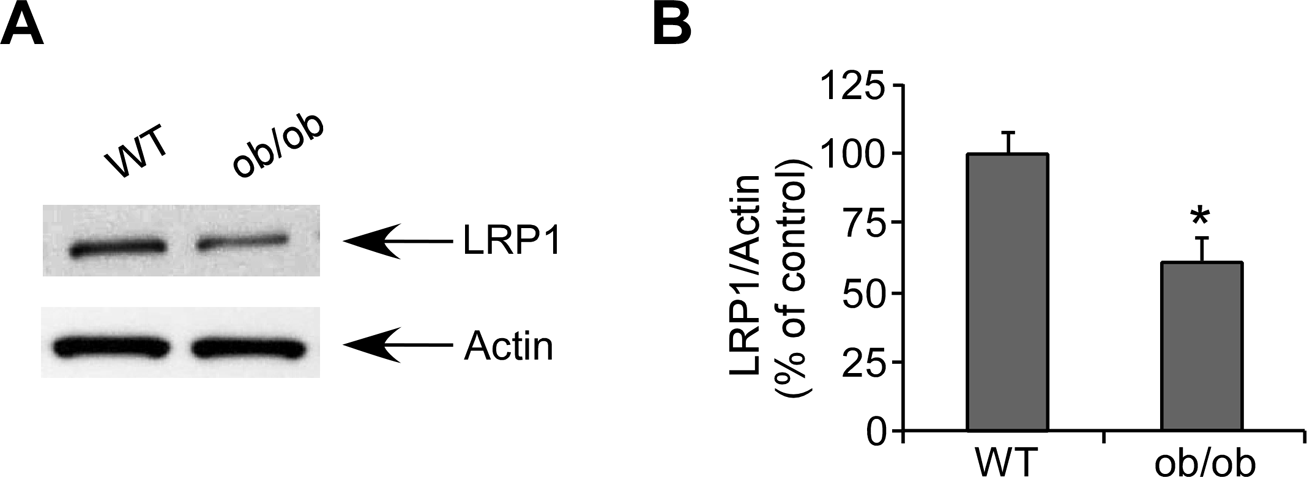

Supplement: Figure S11 — LRP1 expression levels are significantly decreased in ob/ob mice. (A) LRP1 expression levels in the hypothalamus were compared between WT and ob/ob mice at 4 mo of age by Western blotting. LRP1 expression levels were significantly decreased in the hypothalamus of ob/ob mice. (B) Densitometric analysis of Western blot samples (n = 4, *p<0.05). (0.11 MB TIF) [file pbio.1000575.s011.tif]

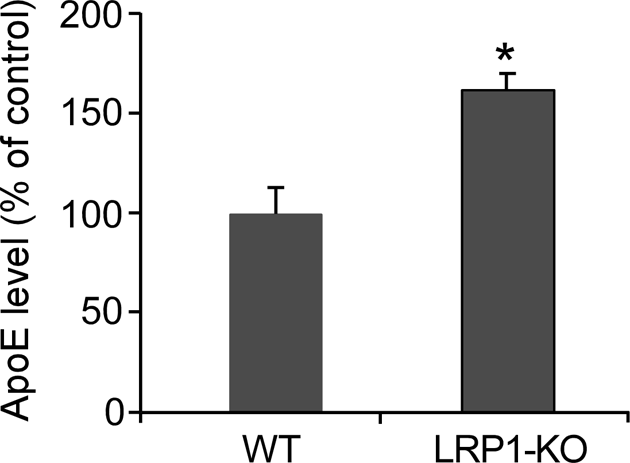

Supplement: Figure S12 — LRP1 deletion increases apoE levels in the hypothalamus. ApoE levels were measured in hypothalamic lysates of 13-mo-old WT and LRP1-KO mice (n = 5), normalized against total protein, and plotted as a percentage of WT controls. *p<0.05. (0.05 MB TIF) [file pbio.1000575.s012.tif]
