# Supplementary material for: Lipoprotein Receptor LRP1 Regulates Leptin Signaling and Energy Homeostasis in the Adult Central Nervous System
Source: PLoS Biol. 2011 Jan 11;9(1):e1000575. doi: 10.1371/journal.pbio.1000575 (PMC3019112; doi:10.1371/journal.pbio.1000575)
Supplement: Text S1 — Supplementary experimental procedures. (0.04 MB DOC) [file pbio.1000575.s013.doc]

**Supplementary Experimental Procedures**

**Lentivirus injection.** For injection of lentivirus into the hypothalamus, male *Lrp1* floxp mice at 8-10 weeks of age (n = 6-14 each group) were anesthetized with 2.5% avertin and injected with lentivirus expressing either Cre or GFP using a stereotaxic table (David Kopf Instruments). All surgical procedures were performed under sterile conditions. With the head in the flat-skull position, one microliter of lentiviral-GFP or lentiviral-Cre virus (4 μl, 4.6 x 10^8^ TU/ml) was injected bilaterally over a period of 8 min using a Hamilton syringe and a syringe infusion pump. Stereotaxic coordinates for intra-hypothalamic injection are as follows: Anterior/Posterior axis, 1.82 mm from Bregma to the posterior; Lateral, ± 0.3 mm from midline; Depth, 5.7 mm from surface of the skull. Lentivirus mLRP2 (3 μl, 7.6 x 10^8^ TU/ml) was also injected bilaterally into the ARC. For cortical injection, coordinates were 2.0 mm bregma, ± 1.7 mm lateral and –0.6 mm dorsoventral. The needle was removed after 10 min. After the injection, mice were housed individually with ad libitum access to standard chow and water in individual cages in a temperature-controlled environment with a 12/12 h light/dark cycle. Food intake and body weight were monitored daily. The injection into ARC allowed the lentivirus Cre to infect ARC, the ventromedial hypothalamus (VMH) and an area of the dorsomedial hypothalamus (DMH), but not the paraventricular nucleus (PMN) or the lateral hypothalamic area (LHA). Based on the immunofluorescence staining and Western blotting, two mice in each injection group were removed from the final analysis.

**Overexpression of ObRb in GT1-7 cells**

HA-tagged ObRb cDNA was generously provided by Dr. Yves Rouillé (Institut Pasteur de Lille, France). Cells were nucleofected with 3 ug of ObR plasmid or control vector according to the manufacturer’s recommendations (Amaxa Biosystems, Gaithersburg, MD, USA). Immediately after nucleofection, cells were placed in pre-warmed media and used for experiments 48 hours post-transfection.

**Antibodies**

Anti-HA antibody (clone 16B12) was purchased from Covance; anti-AgRP antibody and anti-α-MSH antibody were purchased from Phoenix Pharmaceuticals.

**Western blotting and autoradiography**

Cell monolayers or brain tissue were lysed on ice in lysis buffer (phosphate-buffered saline containing 1% Triton X-100, 1 mM phenylmethylsulfonyl fluoride and protease inhibitor cocktail from Roche). Protein concentration was determined in each sample using a Protein Assay kit (Bio-Rad). An equal amount of sample protein was used for SDS-PAGE. Immunoreactive bands were visualized by enhanced chemiluminescence and exposure to film. For densiometric analyses, immunoreactive bands were scanned using a Kodak Digital Science DC120 Zoom camera and quantified using Kodak Digital Science image analysis software. Autoradiography of ^125^I-labeled protein was performed with dried polyacrylamide gels using Kodak XAR-5 films. Films were placed at -70°C for 24 hours prior to developing.

**Real-time reverse transcriptase-polymerase chain reaction (RT-PCR)**

Total RNA was isolated from tissues using SV Total RNA Isolation System (Promega) and subjected to DNase I digestion for removal of contaminating genomic DNA. Total RNA was dissolved in nuclease-free water and stored at -80°C. Reverse transcription was performed using SuperScript II RNase H-reverse transcriptase (Invitrogen), and reaction mix subjected to quantitative real-time RT-PCR for detection of NPY, AgRP and actin mRNA. Actin was used as internal control for each specific gene amplification. Relative levels of expression were determined using Bio-Rad iCycler iQ software. The real-time value for each sample was averaged and compared using the C_T_ method, where the amount of target RNA (2^– ΔΔCT^) was normalized to the endogenous actin reference (ΔC_T_) and related to the amount of target gene in tissue cells, which was set as the calibrator at 1.0.

**Immunofluorescence Staining**

For mouse brain tissue sections, brains were fixed in 4% paraformaldehyde followed by paraffin embedding and tissue sectioning. After deparaffinization, tissue sections were incubated with a LRP1-specific polyclonal antibody and NeuN monoclonal antibody (Chemicon) or GFAP monoclonal antibody (Chemicon) at 4°C overnight. Primary antibody was then visualized using Alexa488-labeled goat anti-mouse secondary antibody and Alexa594-labeled goat anti-rabbit secondary antibody (Invitrogen).

**Animals and tissue preparation**

LRP1 forebrain knockout mice were generated by breeding the *Lrp1* loxP mice (2) with α-calcium-calmodulin-dependent kinase II-driven Cre recombinase mice (8). Age-matched littermates of LRP1-KO (LRP1 forebrain knockout, *Lrp1^flox+/+^/Cre^+/-^*) or WT mice (*Lrp1* flxop littermate control mice, *Lrp1^flox+/+^, Cre^-/-^*) were used in all experiments. Animals were perfused with PBS-heparin (3 units/ml) and brain regions were dissected and kept frozen at -80°C until further analysis. All animal procedures were approved by the Animal Study Committee at Washington University School of Medicine and in accordance with the regulations of the American Association for the Accreditation of Laboratory Animal Care.

**Chemical cross-linking and immunoprecipatation**

Experiments were performed with ^125^I-leptin cross-linked to unlabeled GT1-7 cells. Briefly, cells were seeded into 6-well plates and were ~80% confluent on the day of experiments. Assay buffer (Dulbecco’s minimal Eagle’s medium containing 0.6% BSA) containing 5 nM ^125^I-leptin was added to cell monolayers, followed by incubation for 1 h at 4°C. After ligand binding at 4°C, cell monolayers were washed three times with PBSc (PBS supplemented with 1 mM CaCl_2_, 0.5 mM MgC1_2_) and incubated with PBSc containing 0.5 mM water-soluble, thio-cleavable crosslinker dithiobis (sulfosuccinimidylpropionate) (DTSSP) (Pierce). After 30 min at 4°C, the crosslinking reaction was quenched by washing two times with Tris-buffered saline. Cells were then solubilized in PBSc containing 1% Triton X-100 (vol/vol), 1 mM phenylmethylsulfonyl fluoride and protease inhibitor cocktail (Roche) for 30 min at 4°C and used for immunoprecipitation.

Cell monolayers or brain tissue were lysed on ice in lysis buffer (phosphate-buffered saline containing 1% Triton X-100, 1 mM phenylmethylsulfonyl fluoride and protease inhibitor cocktail from Roche) and mixed with PBS containing 1% Triton X-100, 0.5% sodium deoxycholate, 1% SDS, 0.5% bovine serum albumin. Primary antibody was added, and samples were mixed overnight at 4^o^C followed by incubation for 1 hour with 50 μl of protein A-agarose beads. After washing, immunoprecipitates were released from beads by boiling for 5 minutes in Laemmli sample buffer and analyzed by SDS-PAGE.

**β-Gal staining**

Mice were sacrificed with 2.5% avertin and perfused transcardially with PBS solution followed by 2% PFA/0.5% glutaraldehyde. Brains were embedded in 4% agarose, and 200 μm coronal sections were obtained. Sections were stained for LacZ for 8 hours at 37°C in 1 ml X-gal/Fe cyanide solution. After staining, sections were washed three times with PBS followed by overnight post fixation and visualized using light microscopy.

**ApoE ELISA**

The sandwich ELISA for mouse apoE has been described previously (30). Briefly, 96-well plates were coated overnight with apoE antibody (WU E4), blocked with 1% milk in PBS. Brain samples were diluted in 0.1% BSA, 0.025% Tween-20 in PBS. Following sample incubation, 3 μg/well of biotinylated goat anti-apoE (Calbiochem) was added. After incubation with the secondary antibody, poly-horseradish peroxidase streptavidin (Pierce) was added at 1:6000 dilution and incubated. The plate was then developed with tetramethylbenzidine (Sigma), and read at 650 nm with a Biotek 600 plate reader (Bio-Tek Instruments).
