# Supplementary figures and images for: Correction: Lipoprotein Receptor LRP1 Regulates Leptin Signaling and Energy Homeostasis in the Adult Central Nervous System
Source: PLoS Biol. 2019 Jun 4;17(6):e3000310. doi: 10.1371/journal.pbio.3000310 (PMC6548348; doi:10.1371/journal.pbio.3000310)

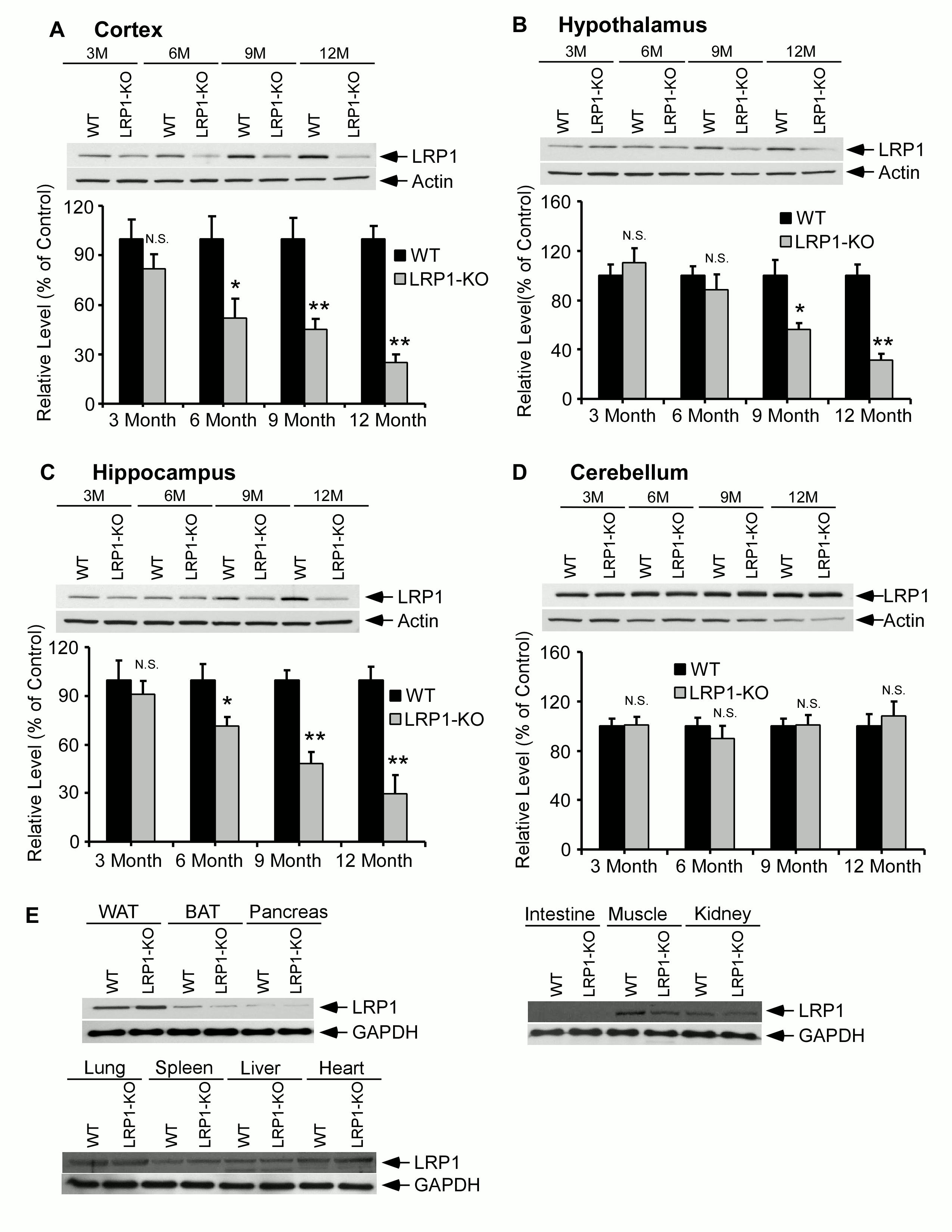

Supplement: S1 Fig — (A–D) LRP1 expression levels were compared between LRP1-KO (Lrp1flox+/+/Cre+/−, LRP1 knockout) and WT (Lrp1flox+/+/Cre−/−, Lrp1 floxP littermate control) mice at 3, 6, 9, and 12 mo of age by Western blotting. Densitometric analysis of Western blots from multiple samples (n = 4) indicated that LRP1 expression was significantly reduced in an age-dependent manner in the cortex (A), hypothalamus (B), and hippocampus (C), but not in the cerebellum (D) of LRP1-KO mice. *p<0.05; **p<0.01; N.S., not significant. For Panels A–D, data are presented as mean ± s.e.m. (E) LRP1 expression levels in selected peripheral tissues were compared between LRP1-KO and WT mice at 12 mo of age via Western blotting. LRP1 expression levels were not significantly altered in white adipose tissue, brown adipose tissue, pancreas, lung, spleen, liver, heart, intestine, muscle, or kidney of LRP1-KO mice. (TIF) [file pbio.3000310.s001.tif]
