# Supplementary material for: Expression of Concern: Lipoprotein Receptor LRP1 Regulates Leptin Signaling and Energy Homeostasis in the Adult Central Nervous System
Source: PLoS Biol. 2025 Dec 1;23(12):e3003528. doi: 10.1371/journal.pbio.3003528 (PMC12668545; doi:10.1371/journal.pbio.3003528)
Supplement: S1 File — (ZIP) [file pbio.3003528.s001.zip › Figure S1.pptx]

## Slide 1
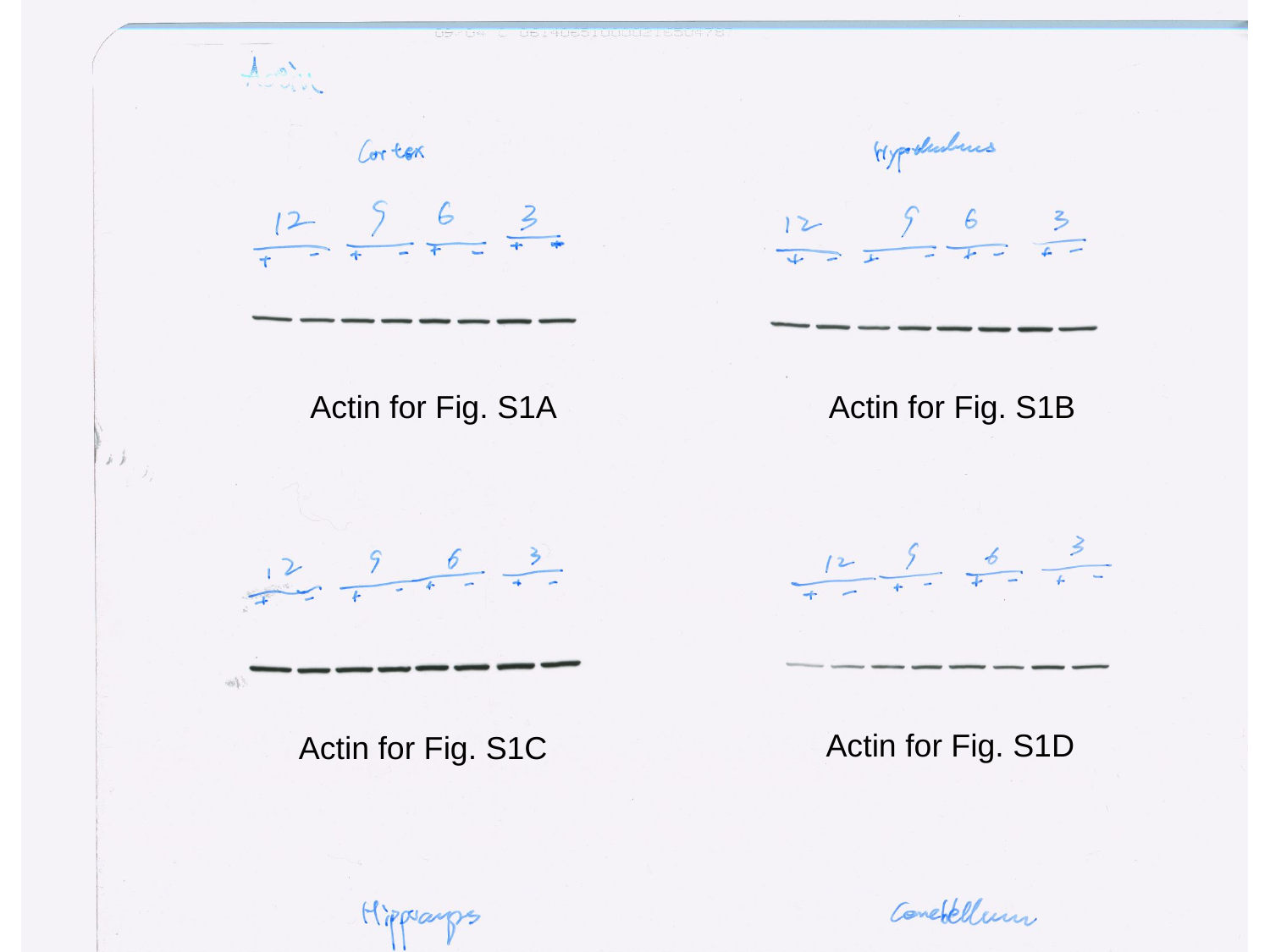

Actin for Fig. S1A
Actin for Fig. S1B
Actin for Fig. S1D
Actin for Fig. S1C

## Slide 2
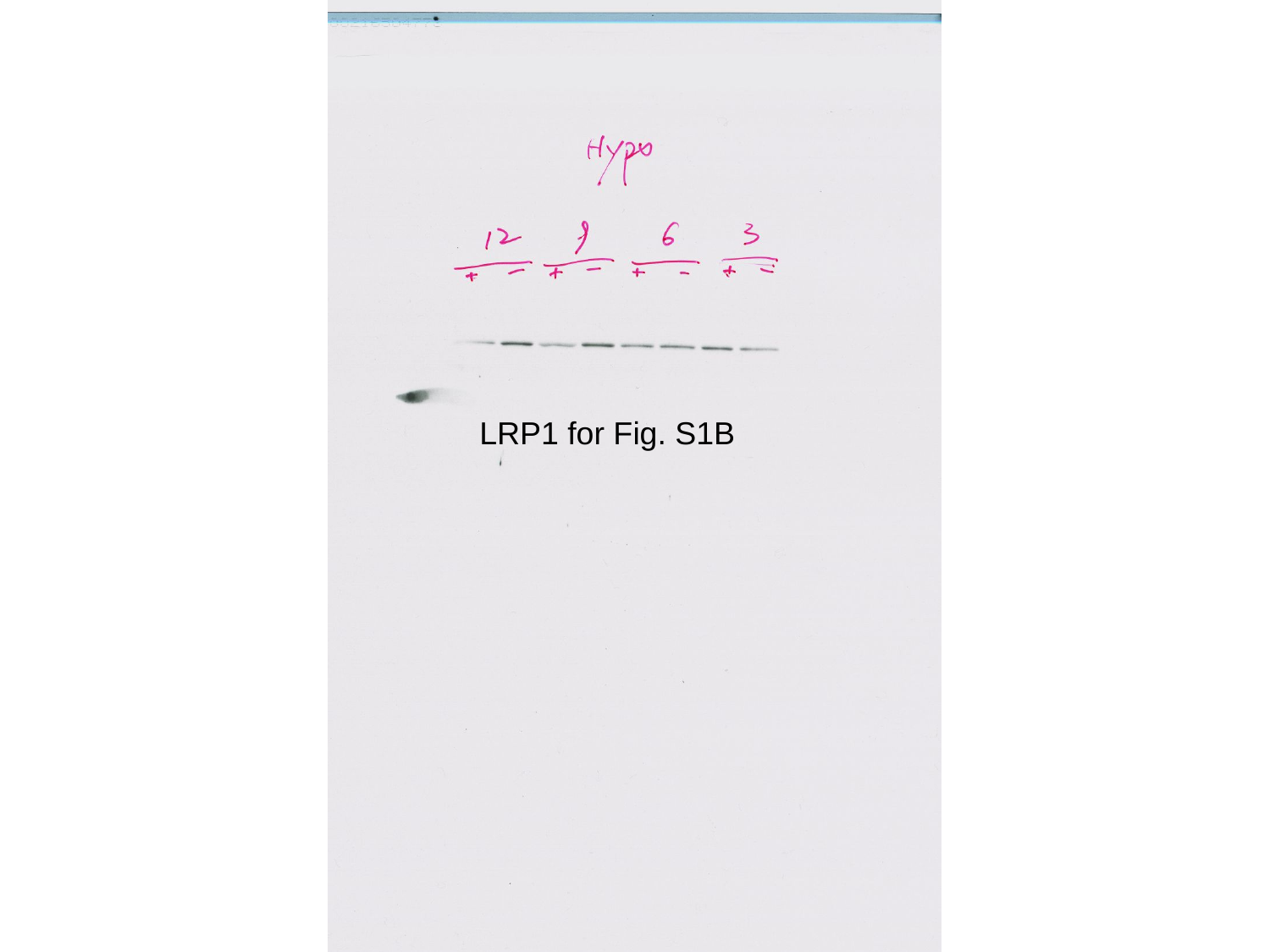

LRP1 for Fig. S1B

## Slide 3
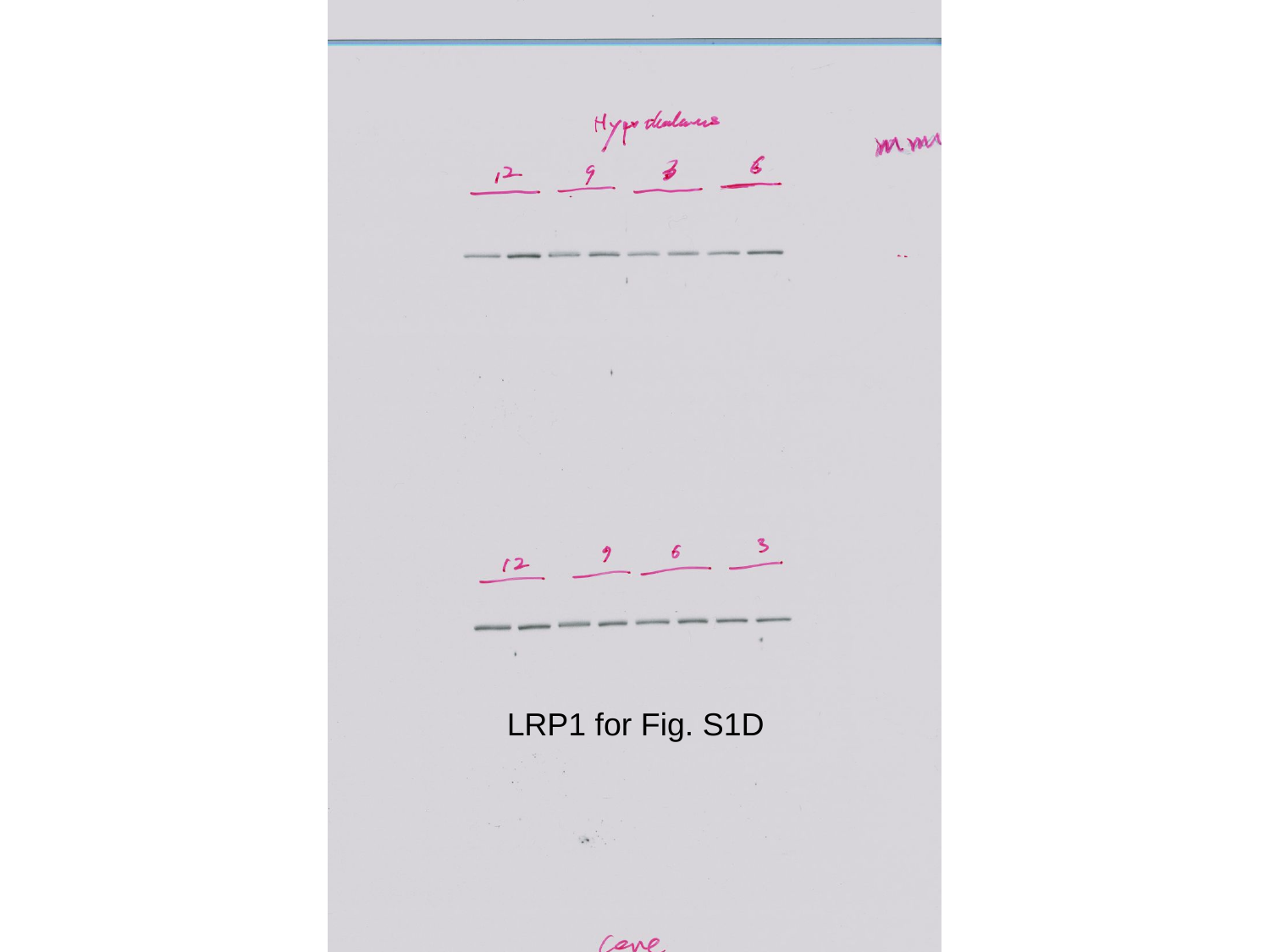

LRP1 for Fig. S1D
