# Supplementary figures and images for: Expression of Concern: Lipoprotein Receptor LRP1 Regulates Leptin Signaling and Energy Homeostasis in the Adult Central Nervous System
Source: PLoS Biol. 2025 Dec 1;23(12):e3003528. doi: 10.1371/journal.pbio.3003528 (PMC12668545; doi:10.1371/journal.pbio.3003528)

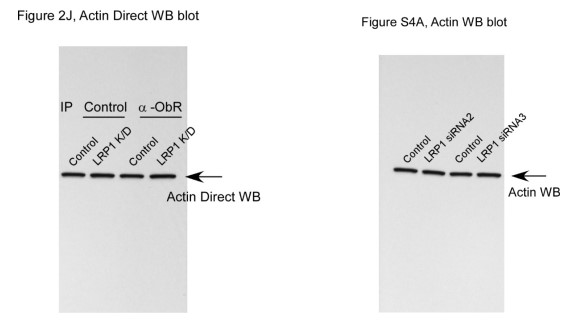

Supplement: S1 File — (ZIP) [file pbio.3003528.s001.zip › Fig 2J Actin Direct WB blot and Fig S4A Actin WB blot.jpg]

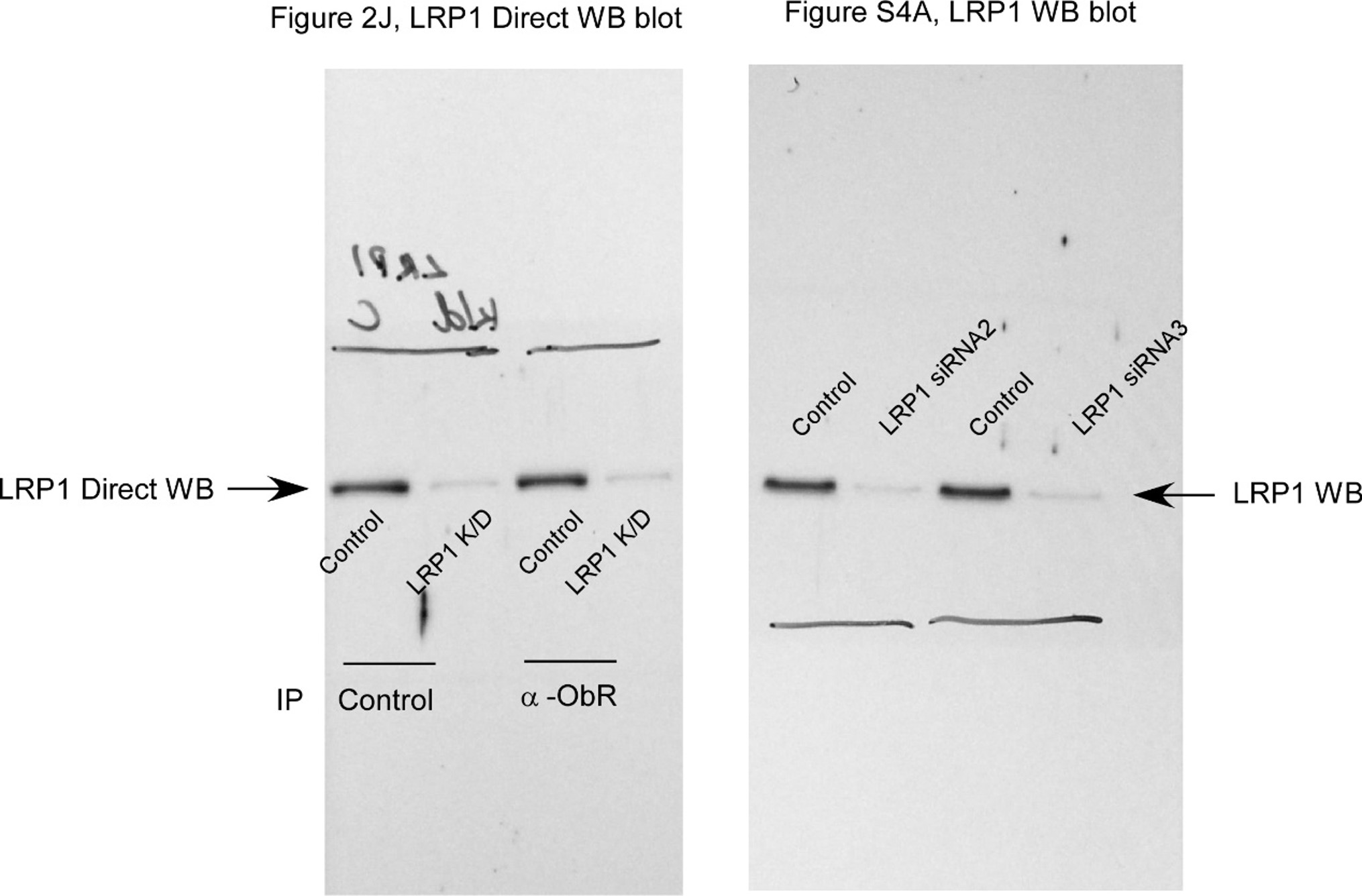

Supplement: S1 File — (ZIP) [file pbio.3003528.s001.zip › Fig 2J LRP1 Direct WB blot and Fig S4A LRP1 WB blot.jpg]

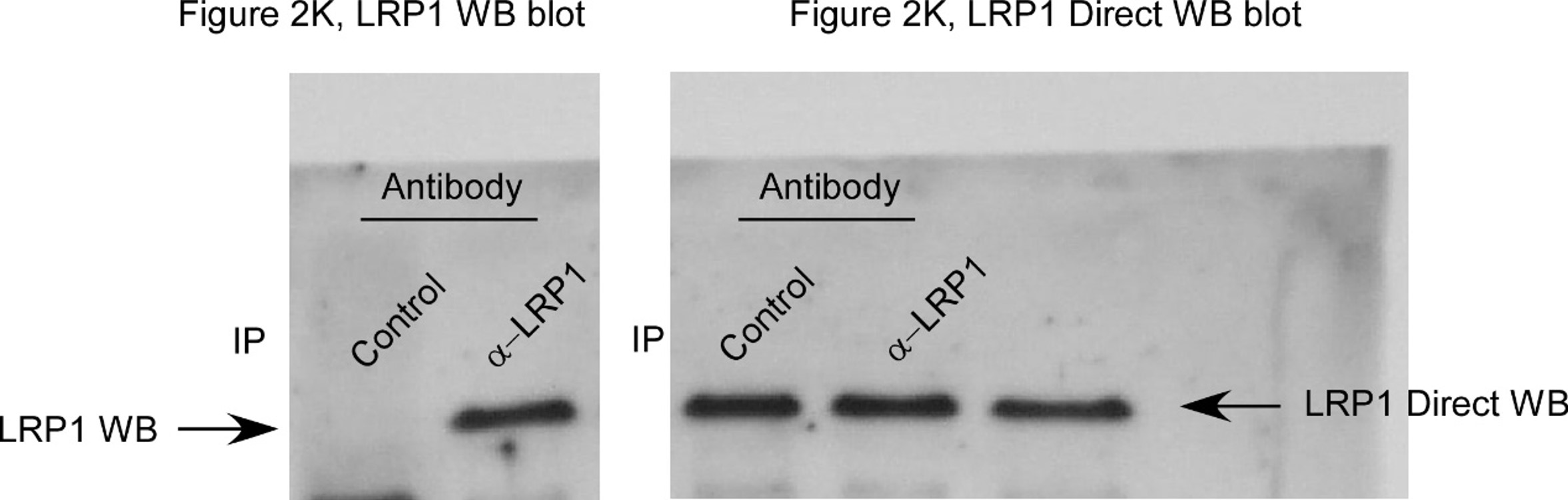

Supplement: S1 File — (ZIP) [file pbio.3003528.s001.zip › Fig 2K LRP1 WB blot and Figure 2K LRP1 Direct WB blot.jpg]

Figure 2H, ObR blot  
Figure 2H, Stat3 blot

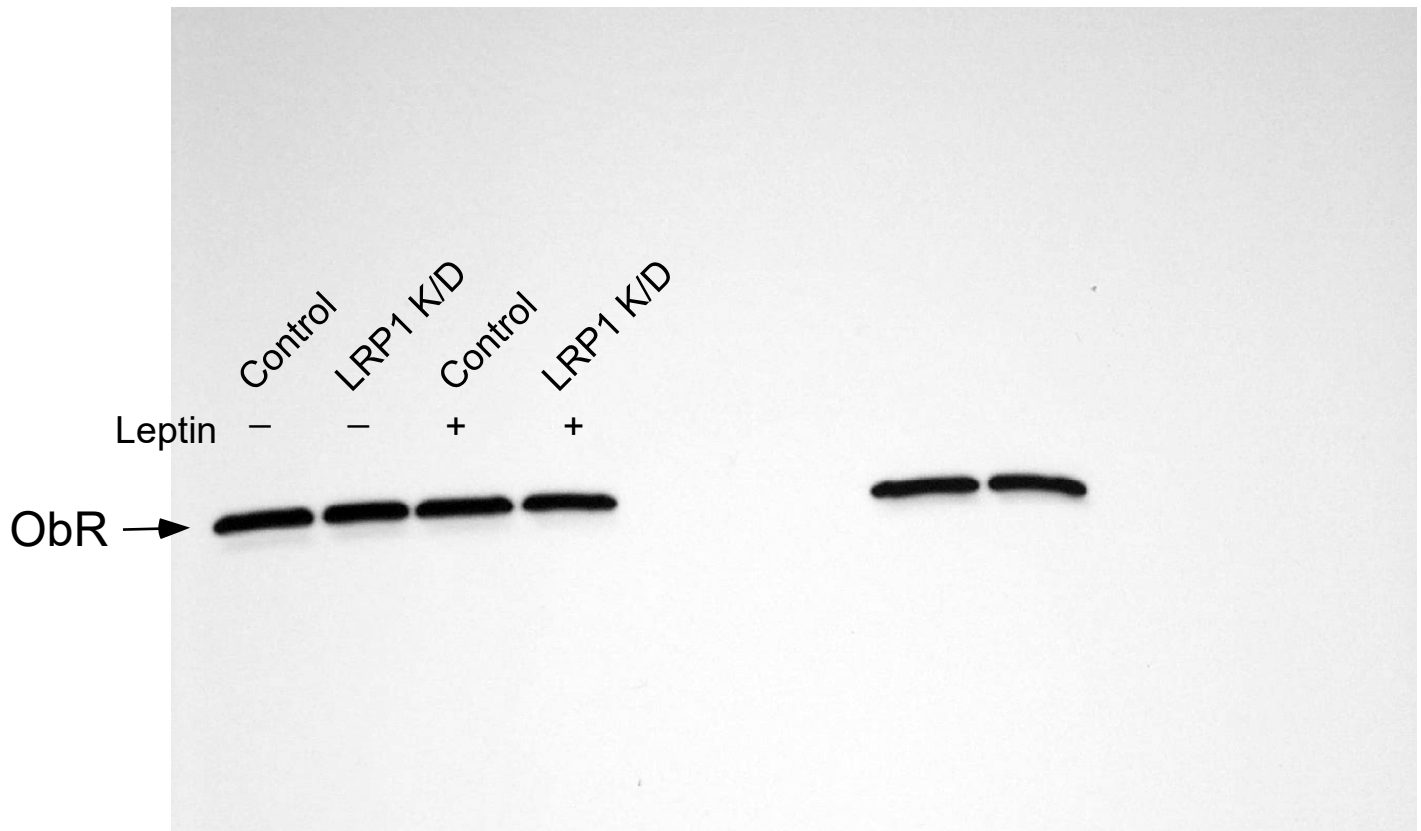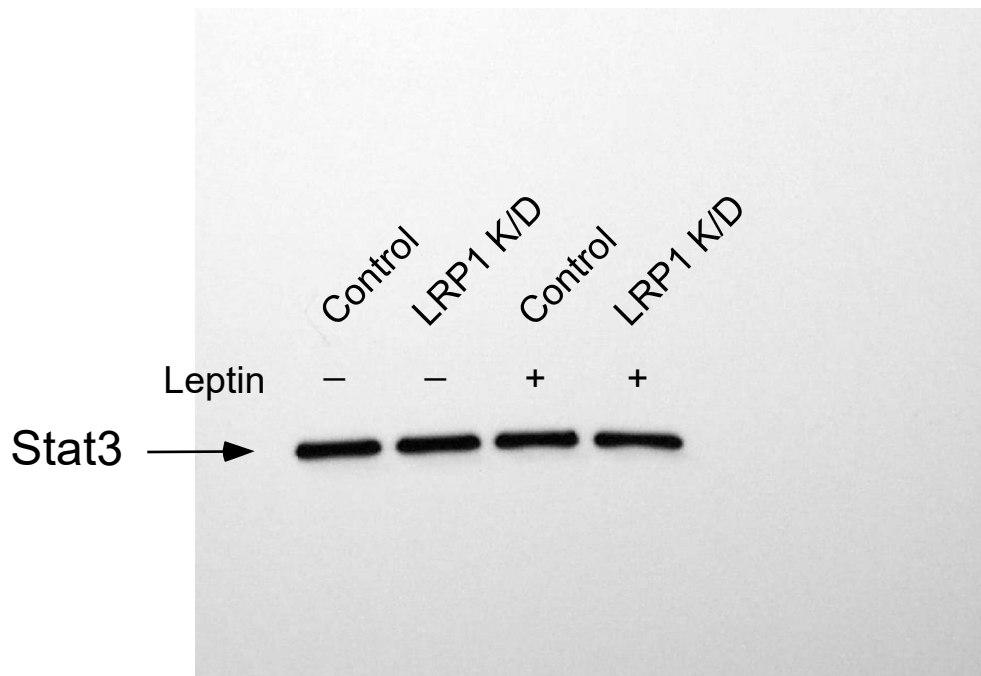

Figure 2H, Actin blot

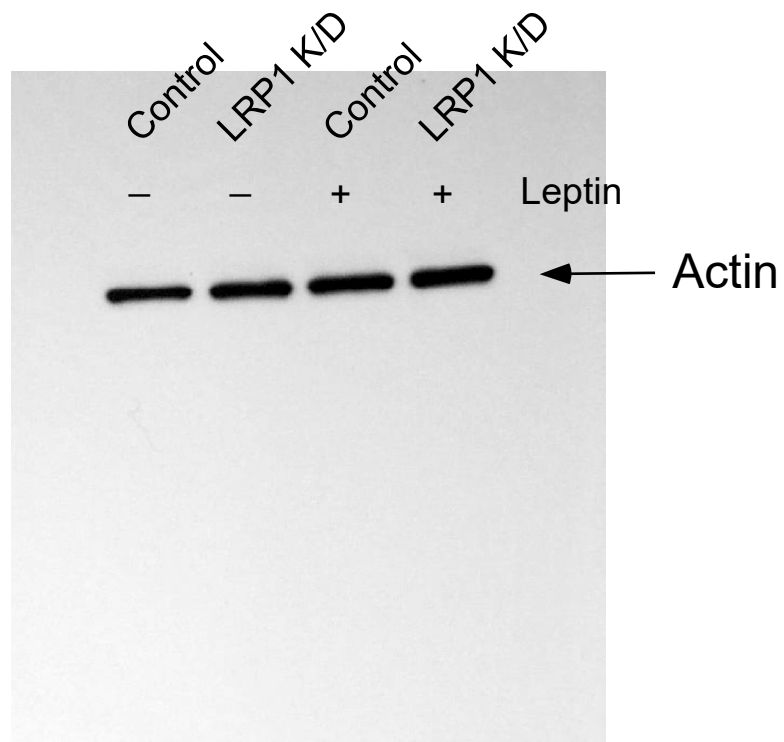

Supplement: S1 File — (ZIP) [file pbio.3003528.s001.zip › Figure 2H.pdf]

Figure 2J, JAK2 Direct WB blot

Figure 2J, ObR Direct WB blot

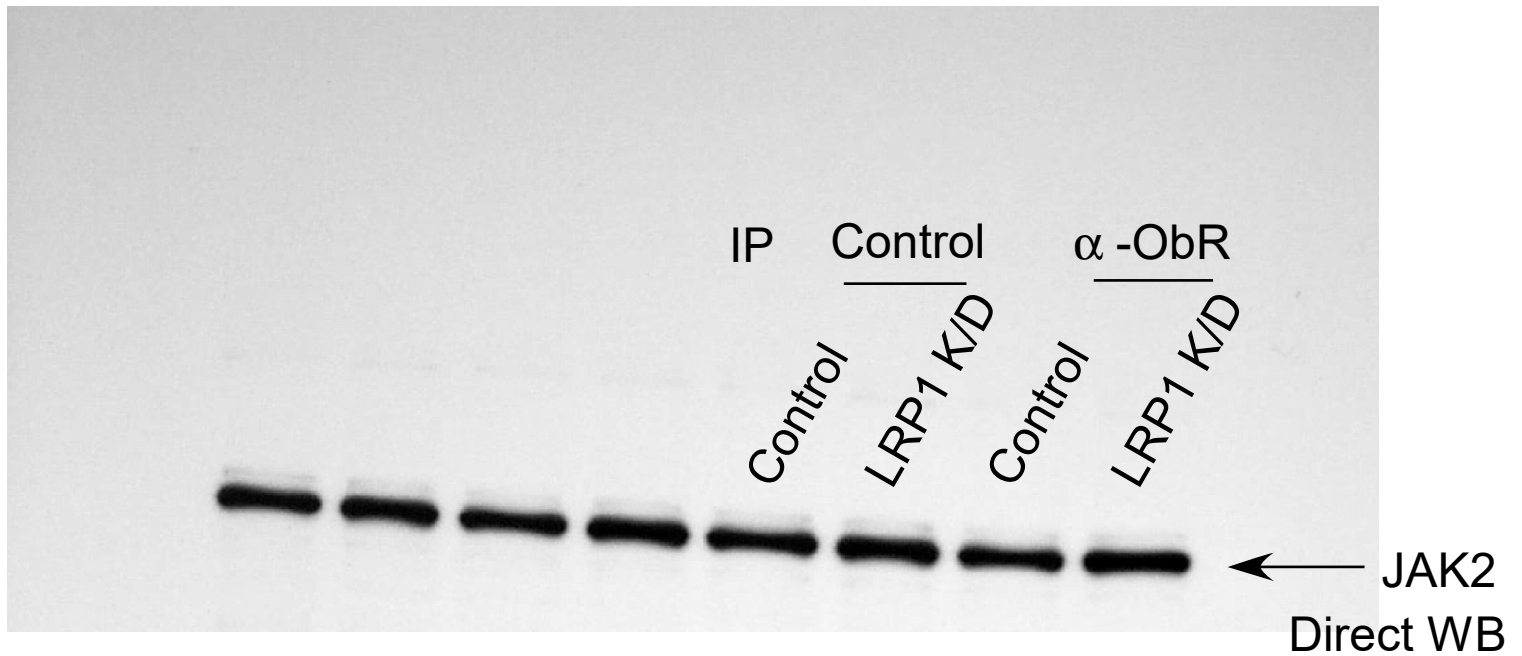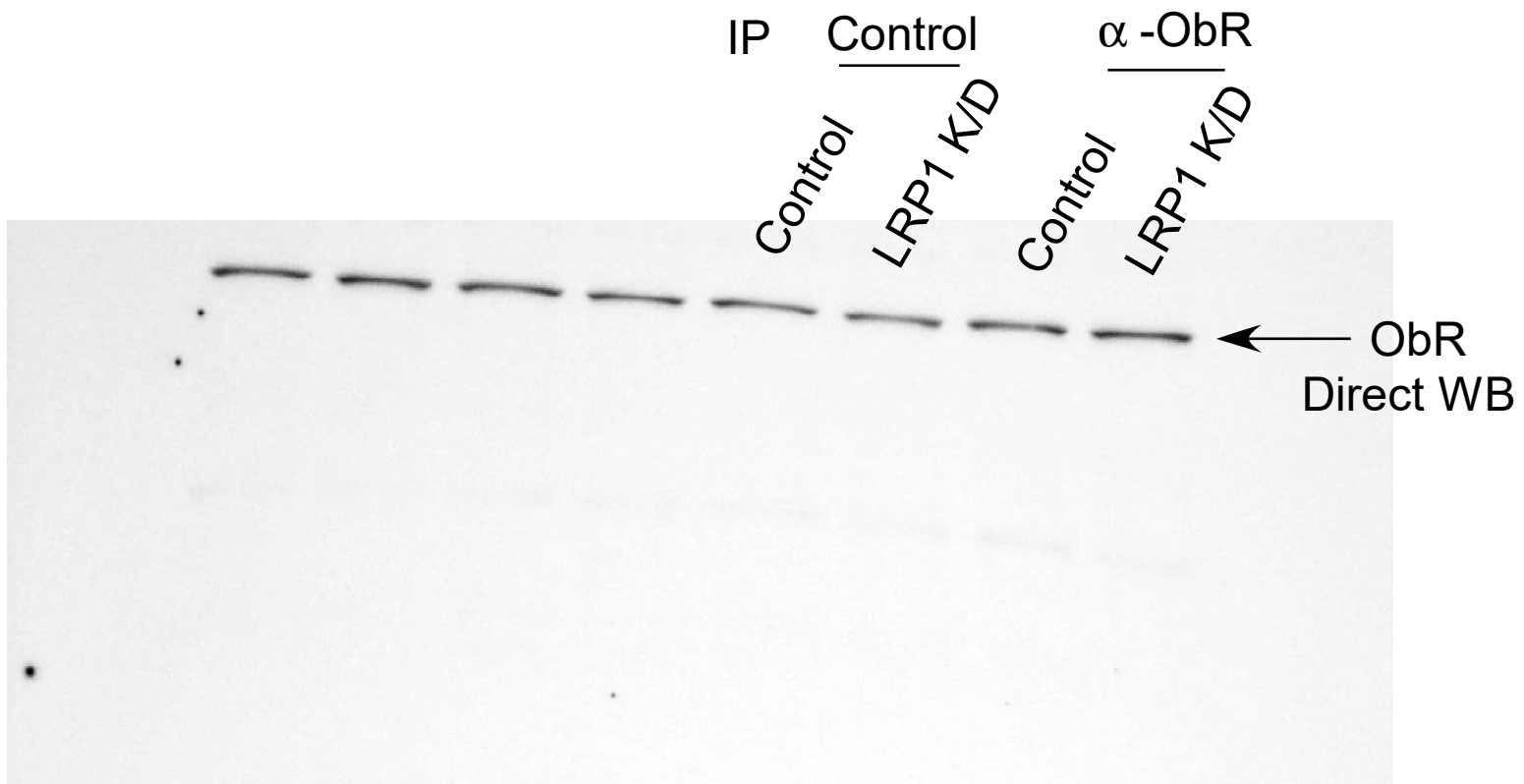

Figure 2J, LRP1 Direct WB blot

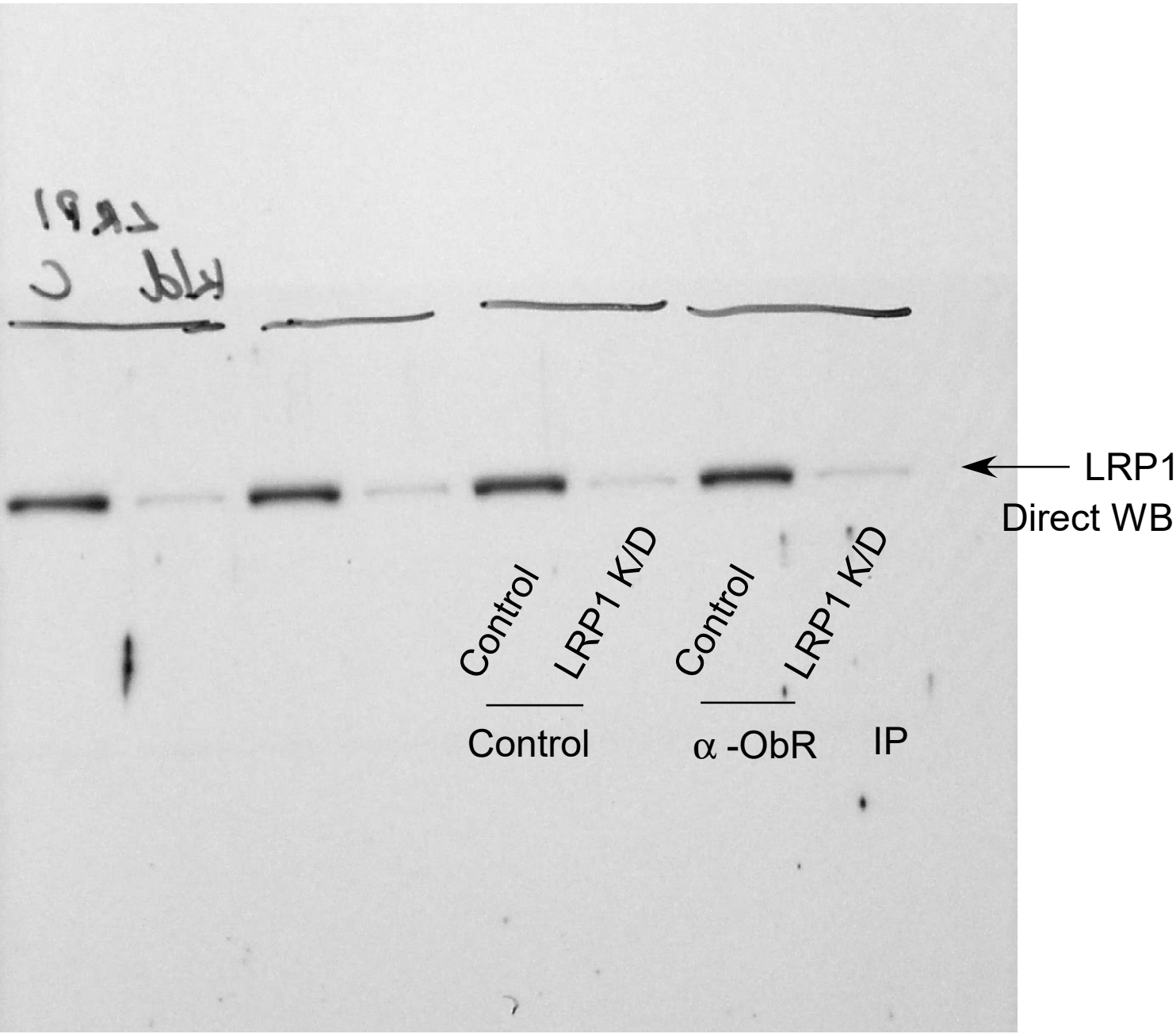

Supplement: S1 File — (ZIP) [file pbio.3003528.s001.zip › Figure 2J.pdf]

Figure S6A, Actin blot

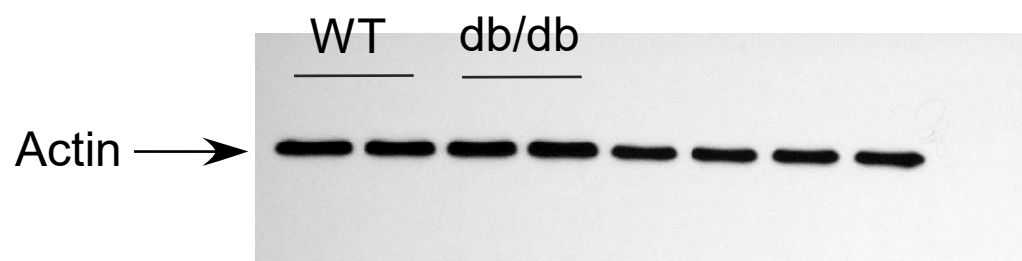

Supplement: S1 File — (ZIP) [file pbio.3003528.s001.zip › Figure S6A.pdf]

Figure S6C, ObR Direct WB blot  
Figure S6C, LRP1 Direct WB blot

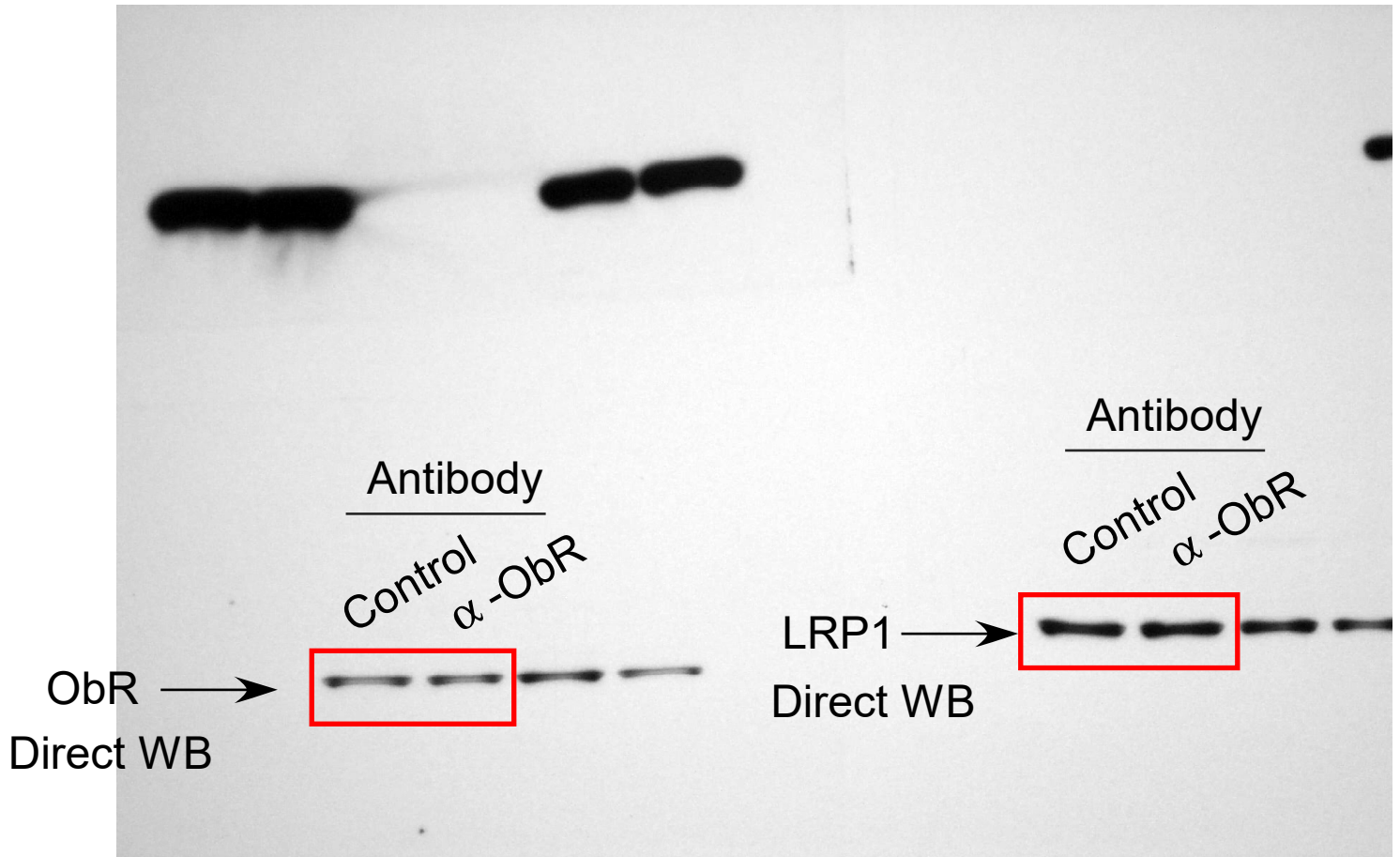

Supplement: S1 File — (ZIP) [file pbio.3003528.s001.zip › Figure S6C.pdf]

Figure S6D, JAK2 WB blot  
Figure S6D, JAK2 Direct WB blot  
(repeat experiment for Figure S6D)

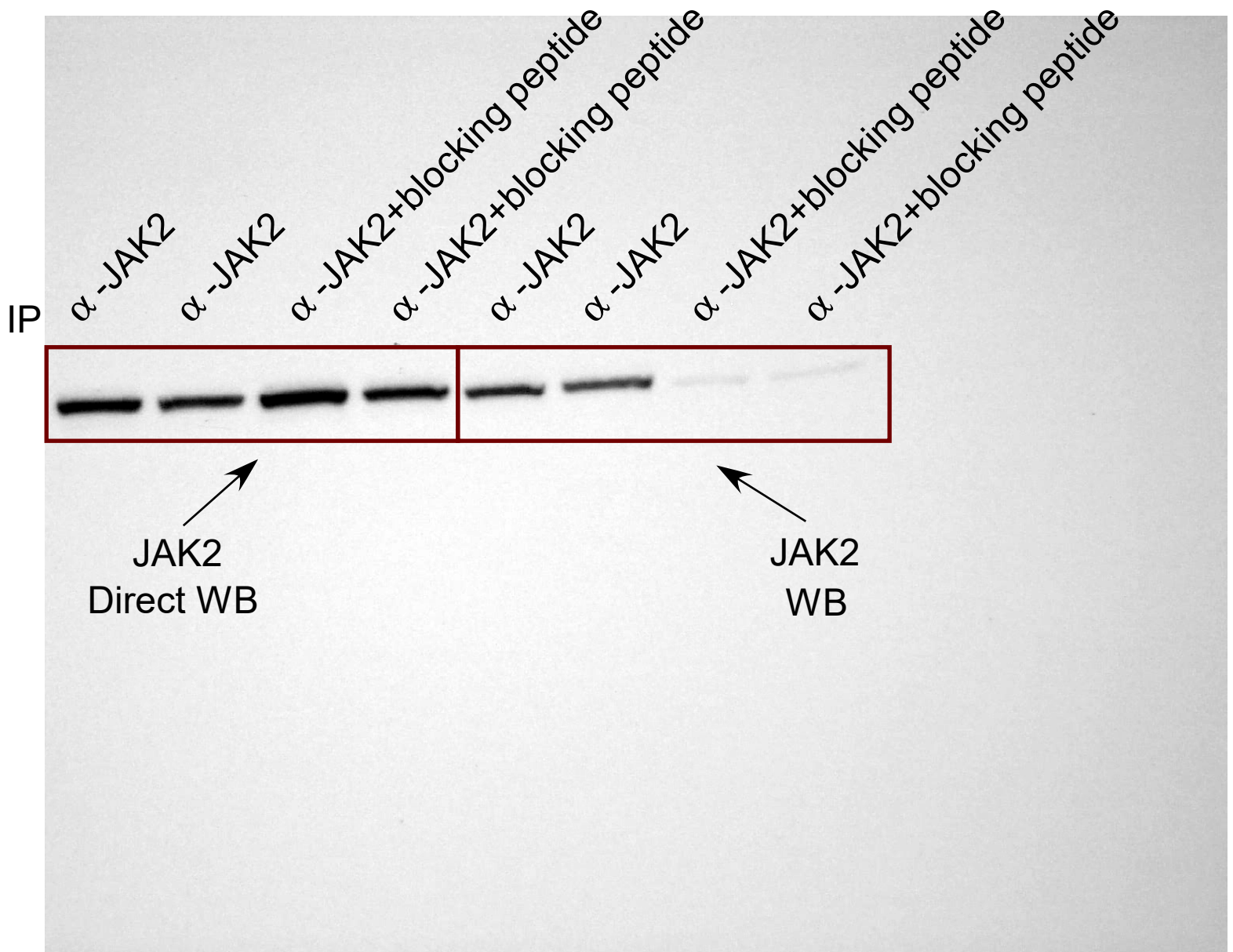

Supplement: S1 File — (ZIP) [file pbio.3003528.s001.zip › Figure S6D.pdf]

**Figure S8A, blot**

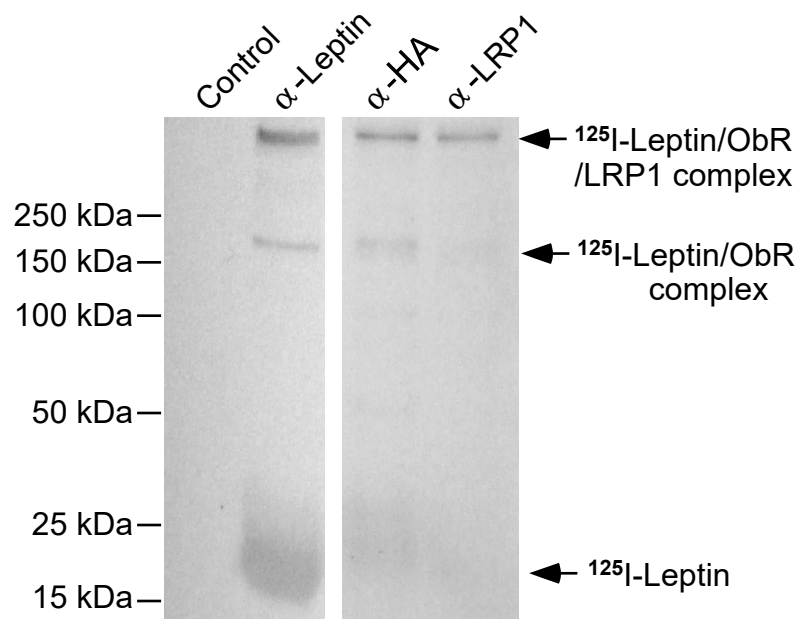

Supplement: S1 File — (ZIP) [file pbio.3003528.s001.zip › Figure S8A.pdf]

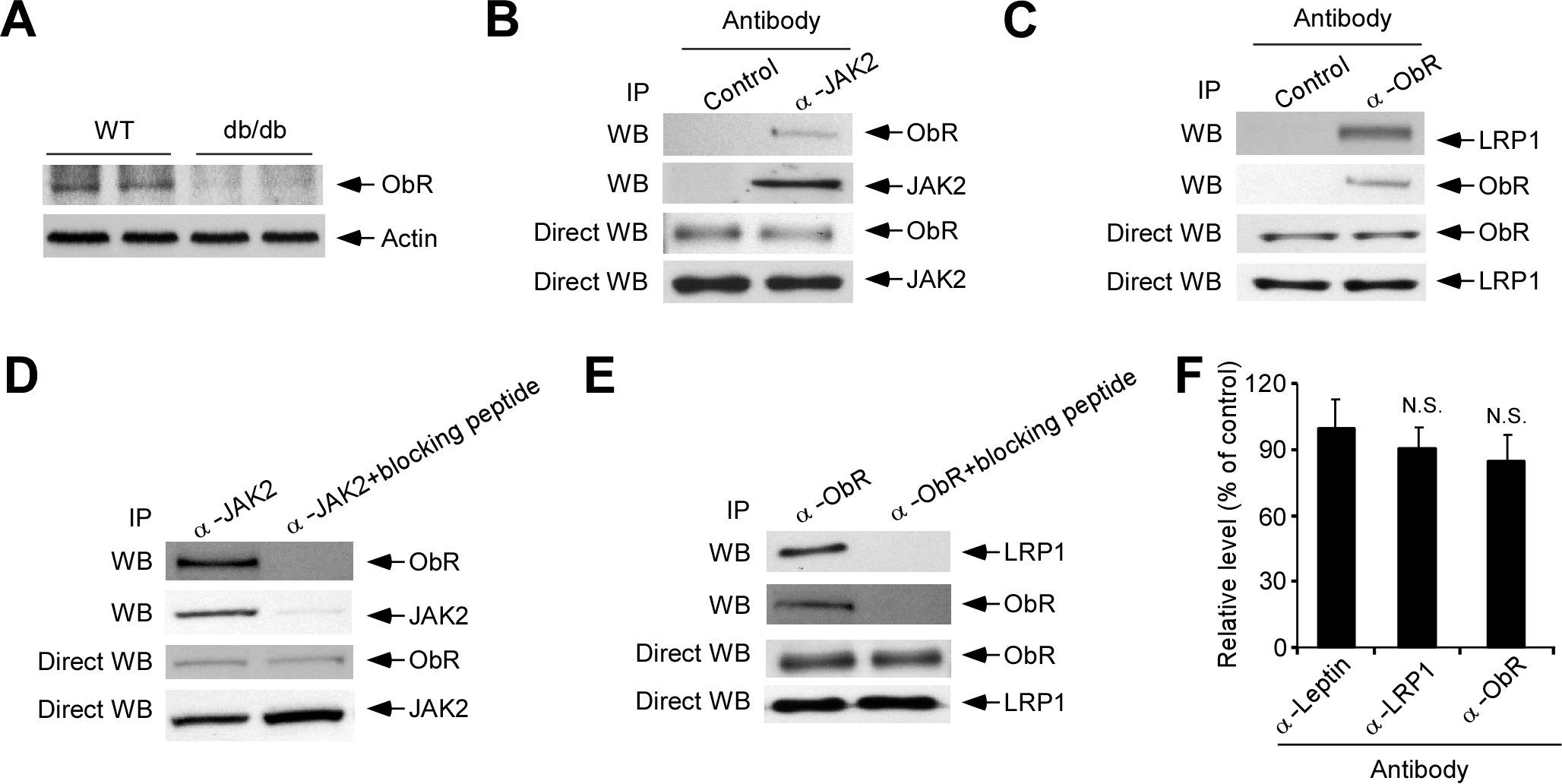

Supplement: Fig S6 — (A) Levels of ObR and actin in the hypothalamus of wild-type and db/db mice (Jackson lab) at 12 wk of age were evaluated by Western blotting. (B) Cellular extracts were prepared from GT1–7 cells and immunoprecipitated with either a control antibody or an anti-JAK2 antibody followed by immunoblotting with an anti-ObR or anti-JAK2 antibody. Extracts were also directly immunoblotted with the ObR antibody and JAK2 antibody. (C) Cellular extracts prepared from GT1–7 cells were immunoprecipitated with either a control antibody or an anti-ObR antibody, followed by immunoblotting with an anti-LRP1 or anti-ObR antibody. Extracts were also directly immunoblotted with the ObR antibody and LRP1 antibody. (D) Extracts were prepared from GT1–7 cells and immunoprecipitated with either an anti-JAK2 antibody or an anti-JAK2 antibody with specific blocking peptide, followed by immunoblotting with an anti-ObR or anti-JAK2 antibody. Extracts were also directly immunoblotted with the ObR antibody and JAK2 antibody. (E) Extracts were prepared from GT1–7 cells and immunoprecipitated with either an anti-ObR antibody or an anti-ObR antibody with specific blocking peptide, followed by immunoblotting with an anti-LRP1 or anti-ObR antibody. Extracts were also directly immunoblotted with the ObR antibody and LRP1 antibody. (F) Densitometric quantification of immunoreactive bands from Figure 2L was performed as described in Materials and Methods (n = 4, N.S., not significant). (TIF) [file pbio.3003528.s002.tif]
